# Advancing sustainable water treatment strategies: harnessing magnetite-based photocatalysts and techno-economic analysis for enhanced wastewater management in the context of SDGs

**DOI:** 10.1007/s11356-024-32680-9

**Published:** 2024-03-12

**Authors:** Khumbolake Faith Ngulube, Amal Abdelhaleem, Ahmed I. Osman, Lai Peng, Mahmoud Nasr

**Affiliations:** 1 Environmental Engineering Department, Egypt-Japan University of Science and Technology (E-JUST), Alexandria, 21934 Egypt; 2 School of Chemistry and Chemical Engineering, David Keir Building, Queen’s University Belfast, Stranmillis Road, Belfast, Northern Ireland BT9 5AG UK; 3 School of Resources and Environmental Engineering, Wuhan University of Technology, Luoshi Road 122, Wuhan, 430070 China; 4 Sanitary Engineering Department, Faculty of Engineering, Alexandria University, Alexandria, 21544 Egypt

**Keywords:** Magnetite-based photocatalysts, Advanced oxidation processes, Dye photodegradation, Techno-economic analysis, Nanocomposite materials, Wastewater treatment

## Abstract

Herein, we explore the holistic integration of magnetite-based photocatalysts and techno-economic analysis (TEA) as a sustainable approach in wastewater treatment aligned with the Sustainable Development Goals (SDGs). While considerable attention has been devoted to photocatalytic dye degradation, the nexus between these processes and techno-economic considerations remains relatively unexplored. The review comprehensively examines the fundamental characteristics of magnetite-based photocatalysts, encompassing synthesis methods, composition, and unique properties. It investigates their efficacy in photocatalytic degradation, addressing homogeneous and heterogeneous aspects while discussing strategies to optimize photodegradation efficiency, including curbing electron–hole recombination and mitigating scavenging effects and interference by ions and humic acid. Moreover, the management aspects of magnetite-based photocatalysts are examined, focusing on their reusability and regeneration post-dye removal, along with the potential for reusing treated wastewater in relevant industrial applications. From a techno-economic perspective, the study evaluates the financial feasibility of deploying magnetite-based photocatalysts in wastewater treatment, correlating reduced pollution and the marketing of treated water with social, economic, and environmental objectives. By advocating the integration of magnetite-based photocatalysts and TEA, this paper contributes insights into scalable and profitable sustainable wastewater treatment practices. It underscores the alignment of these practices with SDGs, emphasizing a comprehensive and holistic approach to managing wastewater in ways that meet environmental, economic, and societal objectives.

**Graphical Abstract:**

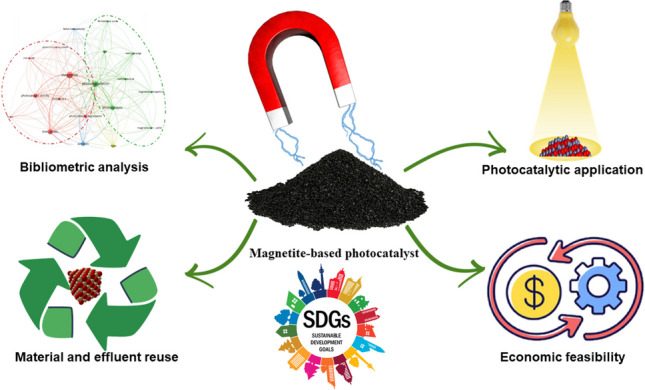

**Supplementary Information:**

The online version contains supplementary material available at 10.1007/s11356-024-32680-9.

## Introduction

Dyes serve as essential components in a wide array of commercial industries, including textiles, clothing, cosmetics, food, beverages, and pharmaceuticals. Regrettably, the textile manufacturing sector alone releases over 200,000 tonnes of dyes annually into aquatic ecosystems, triggering grave environmental concerns (Abdelhaleem et al. 2022). The consequences of dye-related pollution extend beyond environmental disturbances. Human health is risked through direct skin contact or oral ingestion, as dye residues may accumulate in biological tissues and cells. The introduction of dyes into aquatic systems can engender multiple issues, spanning from altered water transparency to the emergence of toxicity, carcinogenicity, and mutagenicity (Kiron 2021a). Such pollution takes a toll on surface water ecosystems, impairing the photosynthetic capabilities of aquatic plants due to diminished sunlight penetration (Lellis et al. 2019). The implications are not confined to aquatic environments; the chemical residues from textile industrial effluents can disrupt the nutrient balance in marine ecosystems, affecting the entire human food chain, especially fish and seafood products (Hussain et al. 2020). It is imperative to recognize that some dye molecules break down into complex chemical compounds and persistent pollutants, further deteriorating natural water resources, including ponds, lakes, streams, and rivers (Pipil et al. 2022). From a human health perspective, the presence of high dye concentrations can inflict harm on vital organs such as the liver, kidneys, skin, and nervous system. Given the multifaceted and far-reaching negative impacts, the urgent need arises to curtail dye contamination within industrial effluents to safeguard environmental and human well-being.

In recent years, advanced oxidation processes (AOPs) have emerged as a promising alternative for the decolorization and mineralization of dye-rich wastewater, offering several advantages over conventional techniques (Tawfik et al. 2022). Conventional methods like biological treatment, adsorption, and coagulation/flocculation often suffer from various shortfalls, such as extended reaction periods, substantial sludge generation, and incomplete decolorization (Alkaim et al. 2014). A key factor in the success of AOPs for dye removal lies in the generation of reactive oxygen species (ROS) from a primary oxidant, such as hydrogen peroxide or ozone, or through reactions involving charge carriers, like electrons (Mourdikoudis et al. 2018). The generated ROS are then able to effectively and non-selectively degrade contaminants such as dyes (Farhadian and Farhadian 2016).

Photocatalysis, a subset of AOPs, involves the activation of a semiconductor material using natural or artificial light sources, typically with energy equal to or higher than the semiconductor’s bandgap energy (Osman et al. 2023). This process is instrumental in the degradation of dyes and can be categorized into two main approaches: heterogeneous photocatalysis: this method involves the use of a nanocatalyst, such as zinc oxide (ZnO) and titanium dioxide (TiO_2_), in conjunction with UV light (Byrne et al. 2015), and homogeneous photocatalytic processes including Fenton and photo-Fenton methods (Moradi et al. 2021).

Recent studies have highlighted the potential of AOPs for the removal of synthetic dyes. These processes efficiently break down complex dyes into simpler organic compounds, offering cost-effective and time-efficient solutions (Ajmal et al. 2014; Bastos-Arrieta et al. 2018; Karim et al. 2022). In a study conducted by Ahmed and Haider (2018), the benefits of heterogeneous photocatalysis for textile effluent treatment were underscored, including (i) heterogeneous photocatalysis can transform dyes into CO_2_ and various inorganic substances; (ii) this process occurs at standard temperature and pressure, enhancing practicality; (iii) importantly, it avoids the excessive generation of sludge, reducing waste concerns; (iv) the process primarily relies on the presence of oxygen and energy from sunlight, making it sustainable and environmentally friendly; and (vi) catalysts can be supported on a range of inert matrices, including glasses, polymers, carbon nanotubes, and graphene oxides. Furthermore, the prepared catalysts can be both cost-effective and environmentally friendly, as they are often cheap, non-toxic, and reusable (Karim et al. 2022), making an environmentally friendly approach for dye removal.

Magnetite (Fe_3_O_4_) emerges as a compelling iron oxide photocatalyst with a unique set of attributes, making it a valuable choice for various applications (Osman et al. 2022). Notably, this photocatalytic material exhibits remarkable magnetic and electrical properties, a narrow band gap, exceptional thermal stability, and biocompatibility (Rajamohan et al. 2017). One of the standout features of Fe_3_O_4_ photocatalysts is their simple recyclability, which significantly curbs the generation of secondary pollutants during the treatment processes (Mishra et al. 2019). However, it is worth noting that pure Fe_3_O_4_ is susceptible to photo-corrosion when exposed to prolonged light irradiation. To counter this limitation, it is often integrated with other semiconducting materials, a strategy that aids in charge carrier conveyance and transport (Weng et al. 2019). This integration prevents the excessive accumulation of charge carriers on the photocatalyst’s surface, enhancing light absorption by shifting the valence band (VB) and conduction band (CB) positions. Given the interplay of cost and efficiency, the utilization of AOPs based on photocatalytic semiconductors in treating textile industrial effluents is a critical area of research. The degradation of dyes using magnetite-related photocatalysts is influenced by several key factors, including solution pH, temperature, pollutant concentrations, irradiation time, and photocatalyst dosage (Abdelhaleem et al. 2022; Manohar et al. 2021). To optimize the efficacy of different magnetite-based photocatalysts, it is essential to define and apply the most suitable operating conditions. These conditions play a pivotal role in achieving higher levels of dye decolorization efficiency, a goal of paramount importance in wastewater treatment processes.

The application of magnetite-based photocatalysis to treat dye-laden wastewater holds immense promise in safeguarding environmental integrity (Moradi et al. 2021). However, in the pursuit of comprehensive sustainability, it is imperative to address certain challenges that align with the economic and social dimensions, guided by the principles of the United Nations’ sustainable development goals (SDGs) (Weng et al. 2019). These 17 SDGs encompass a framework of global objectives adopted by UN member states, aiming to secure global peace and prosperity in the present and future (Hák et al. 2016). Treating effluents from the textile industry to preserve aquatic ecosystems directly correlates with SDG6, “Clean water and sanitation,” and SDG14, “Life below water.” Furthermore, safeguarding human health against the toxicity of textile dyes aligns with the targets of SDG3, “Good health and well-being.”

In addition to these direct associations, the high degradation efficiency and reusability performance of magnetite-based photocatalysts contribute to the reduction of solid waste disposal on land and open-air burning, thus aligning with SDG15, “Life on land.” Moreover, the promotion of innovation and industrialization in photocatalyst synthesis opens new avenues for the creation of environmentally friendly jobs, supporting SDG8, “Decent work and economic growth,” through employment in sustainable projects (Pandit et al. 2023). The synthesis and application of magnetite-based photocatalysts in treating textile effluents offer not only direct but also indirect interlinkages with all 17 SDGs. This interplay underscores the need for a comprehensive exploration of the potential contributions of these photocatalysts to advancing the broader agenda of sustainable development.

To the best of the authors’ knowledge, there is a research gap in addressing the sustainable strategies for dye-laden wastewater treatment. This review paper aims to bridge the gap in existing research by examining the interconnection between photocatalytic dye degradation, techno-economic considerations, photocatalyst management, and their alignment with the social, economic, and environmental dimensions of the SDGs. The primary focus is on the utilization of heterogeneous advanced oxidation processes (AOPs) facilitated by magnetite-based photocatalysts for treating textile industrial effluents, employing a bibliometric approach. The comprehensive scope encompasses factors influencing AOP efficiency, the synthesis and characterization of magnetite-based photocatalysts for dye photodegradation, the economic feasibility of commercial-scale wastewater treatment, strategies for managing treated effluent and spent photocatalysts, and the achievement of relevant SDGs through pollution reduction. The review concludes by addressing challenges, providing recommendations for future research, and underlining the significance of the findings and their potential implications.

## Dyes: types and structures

Different types of dyes have been subjected to the photocatalytic degradation process (Table 1) using magnetite-based photocatalysts. This study highlights the seven types of dyes, namely, azo dyes (Benkhaya et al. 2020; Ogugbue and Sawidis 2011), acid dyes (Kumar et al. 2021; Said et al. 2020), basic dyes (dos Santos et al. 2007; Kiron 2013), vat dyes (dos Santos et al. 2007), reactive dyes (Said et al. 2020), disperse dyes (Kiron 2021b), and sulphur dyes (Chakraborty 2011; El-Sikaily et al. 2012). A detailed discussion of the characteristics of the different dye types is given in the supplementary material.

**Table 1.**
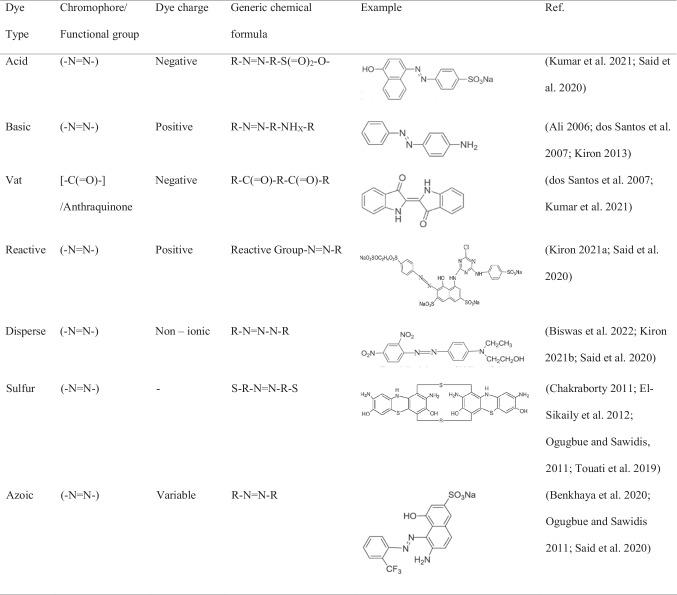
Dye types, their chromophore groups, overall charge, and general chemical formula

## Mechanism and fundamentals of photocatalysis

Photocatalysis obeys a series of photo-activated chemical reactions (i.e., chemical conversion using photonic energy) at a solid surface, usually a semiconductor (e.g., TiO_2_). A photocatalyst material is exposed to light emitting with photons that possess energy equal to or greater than the band-gap energy (Kumari et al. 2023). Subsequently, electron excitation caused an electron to transfer from the valence band (VB) to the conduction band (CB) of the photocatalyst (Eq. 1). This sequence of events generates very reactive electron–hole (*e*^−^/*h*^+^) pairs, forming complex oxidative-reductive chain reactions (Ren et al. 2021).

1 $$\mathrm{Photocatalyst }\,({\text{e}}.{\text{g}}., {{\text{TiO}}}_{2})+hv\to {e}^{-}\left({\text{CB}}\right)+{h}^{+}({\text{VB}})$$

where *e*^−^(CB) and *h*^+^(VB) represent the electrons in the conduction band and the electron vacancy in the valence band, respectively.

**Fig. 1:**
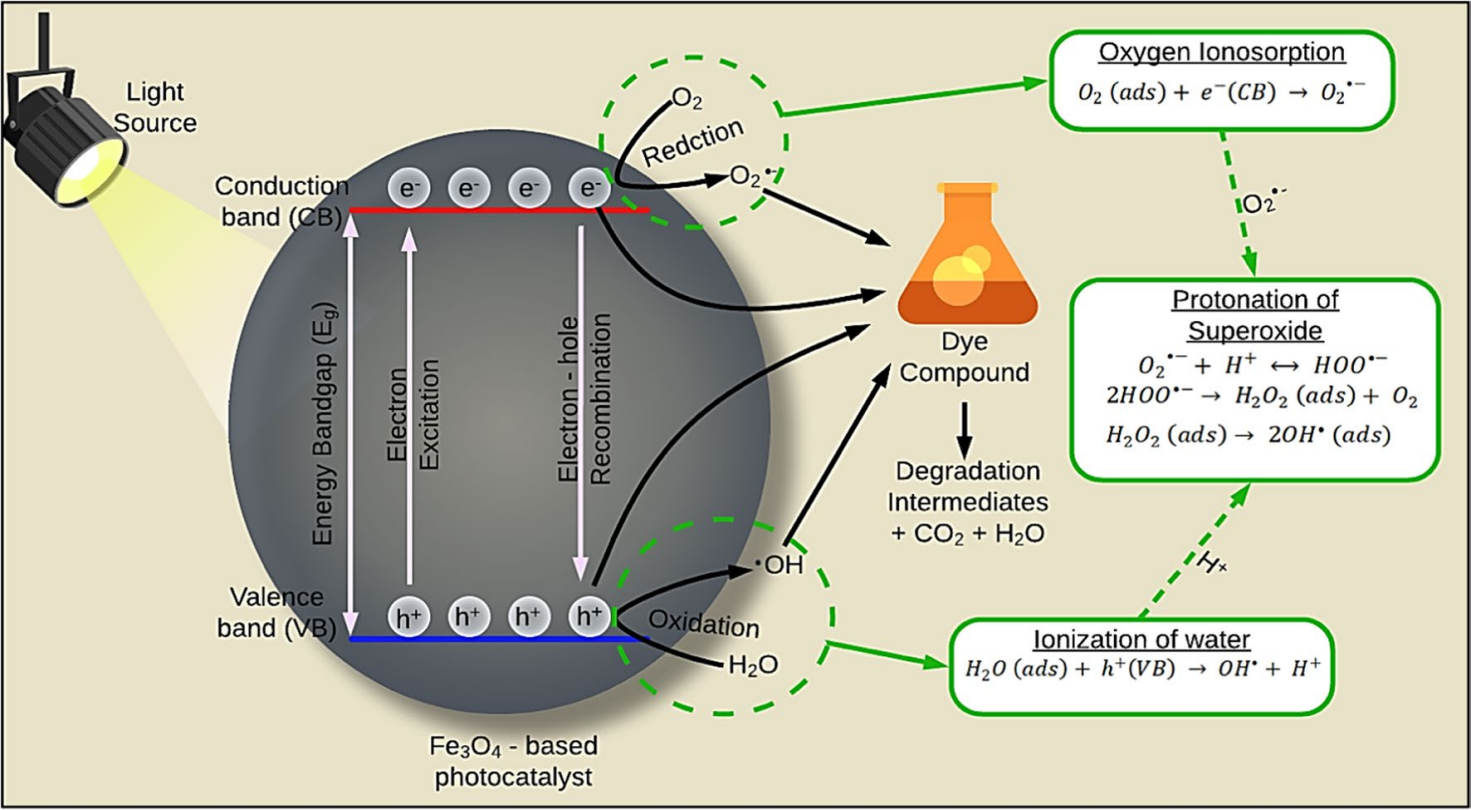
Photocatalytic degradation mechanism for removal of dyes in aqueous media

Following the generation of the *e*^−^/*h*^+^ pairs, either one or two processes might occur, as shown in Fig. 1.

The first pathway is *e*^−^/*h*^+^ recombination (*e*^−^  + *h*^+^  → heat), where the electron in CB surpasses the energy gap and transfers back to the valence band. The gained photon energy is dissipated, and then, the excited electrons become stable to occupy the position of the hole left behind in VB. However, the *e*^−^/*h*^+^ pair recombination should be inhibited using electron scavengers (surface adsorbents) with a larger electron storage capacity. Mixing noble metals, such as silver, gold, and platinum, with the semiconductor photocatalyst is another method to avoid the recombination between *e*^−^ and *h*^+^. The second process occurs when electron donors and/or acceptors are present in the environment surrounding the semiconductor material. The electrons initially accumulate on the photocatalyst surface and commence a cascade of oxidation (oxidation of an electron donor by the holes on the semiconductor surface) and reduction (diffusion of acceptor and reduction by the electrons on the semiconductor surface) reactions (Ajmal et al. 2014). After electron excitation, a series of pollutant degradation mechanisms occurs. Three phases, i.e., (a) water ionization, (b) oxygen ionosorption, and (c) superoxide protonation, could be used to describe the degradation mechanisms (Fig. 1) (Biswas et al. 2022). Ionization of water (Eq. 2) involves the reaction of the generated valence band holes, *h*^+^(VB), with water adsorbed on the photocatalyst surface, producing hydroxyl radicals (^•^OH).

2 $${{\text{H}}}_{2}\mathrm{O }\,\left({\text{ads}}\right)+ {h}^{+}\left(VB\right) {\to }^{\bullet }{\text{OH}}+{H}^{+}$$

The generated ^•^OH radicals are highly reactive species that react with the dyes adsorbed onto the photocatalyst surface, converting them into intermediate species (Ahmed and Haider 2018). The formed intermediate by-products are continuously subjected to this strong oxidant until they are completely broken down into final degradation products such as CO_2_ and H_2_O.

The second set of reactions represents oxygen ionosorption (Eq. 3). In this pathway, the oxygen molecules in the photocatalyst environment are combined with the CB electrons, forming a superoxide radical (O_2_^•−^). This anion of oxygen exhibits strong oxidation of dye molecules adsorbed on the photocatalyst surface continuously, breaking them down into final degradation products. The formation of O_2_^•−^ radicals also assists in reducing the *e*^−^/*h*^+^ pair recombination (Ajmal et al. 2014).

3 $${{\text{O}}}_{2} \left({\text{ads}}\right)+ {e}^{-}\left({\text{CB}}\right) \to {{{\text{O}}}_{2}}^{\bullet -}$$

The third set of reactions is termed as protonation of superoxide radicals. As such, the O_2_^•−^ (superoxide anion) is protonated (reacts with the H^+^ ions) to form hydroperoxyl radicals (HOO^•−^) (Eq. 4) (Ajmal et al. 2014; Hoffmann et al. 1995). As more HOO^•−^ are formed, they tend to react together, forming hydrogen peroxide and oxygen in the solution (Eq. 5). The H_2_O_2_ molecules then proceed to spontaneously dissociate without discernible energy barrier into more ^•^OH (Eq. 6):

4 $${{{\text{O}}}_{2}}^{\bullet -}+{{\text{H}}}^{+}\leftrightarrow {{\text{HOO}}}^{\bullet -}$$

5 $${2{\text{HOO}}}^{\bullet -}\to {{\text{H}}}_{2}{{\text{O}}}_{2}+{{\text{O}}}_{2}$$

6 $${{\text{H}}}_{2}{{\text{O}}}_{2}+hv\to {2}^{\bullet }OH$$

The ^•^OH produced in the previous reactions can react with dye, causing decolouration (Eqs. 7&8):

7 $${}^{\bullet }{\text{OH}}+{\text{dye}}\to \mathrm{intermediates }\to {\text{products}}$$

8 $${\text{Dye}}+{e}^{-}\left({\text{CB}}\right) \to {{\text{dye}}}_{{\text{red}}}$$

In addition to these reactions, *e*^−^ (CB) and *h*^+^ (VB) can react directly with pollutants adsorbed on the photocatalyst surface via the reduction and oxidation reactions, respectively. Furthermore, the final degradation products desorb and detach from the photocatalyst surface into the bulk solution (Ajmal et al. 2014).

## Classification of magnetite-based photocatalysts

**Fig. 2:**
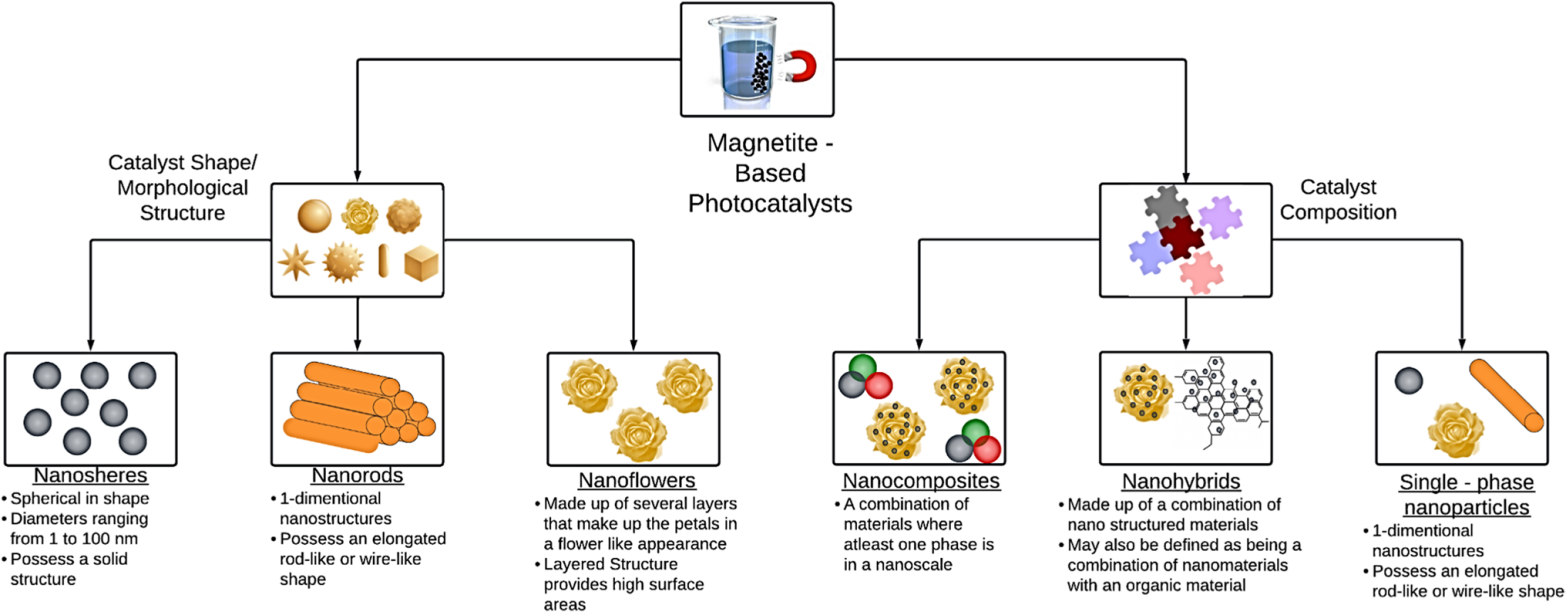
Classification tree of magnetite-based photocatalysts

Magnetite-based photocatalysts, by definition, are photocatalysts that comprise magnetite (Fe_3_O_4_) in their chemical structure. Fe_3_O_4_-contained photocatalysts can be distinguished by their shape (e.g., spheres, rods, and flowers) or composition (composites, hybrids, or single-phase particles) (Fig. 2).

Multipurpose magnetite nanorods were synthesized using a solution-phase method in the existence of polyethylene glycol-1000 (Lian et al. 2003). The synthesized homogenous nanorods exhibited a single crystal structure, and the rod’s average diameter and length were 80 nm and 2 µm, respectively (Lian et al. 2003).

Porous Fe_3_O_4_ nanospheres were synthesized by solvothermal method with FeCl_3_·6H_2_O, polyvinylpyrrolidone (PVP), and sodium acetate as the sole iron supply, the capping agent, and the precipitation agent, respectively (Zhu and Diao 2011). The as-prepared nanospheres had an average diameter of 250 nm due to the agglomeration of smaller Fe_3_O_4_ (Zhu and Diao 2011). The nanosphere material was featured by a highly porous structure with pore sizes in the 3–10 nm range and a surface area of ≈ 47.7 m^2^/g. This feature was suitable for the efficient degradation of xylenol orange (XO) in an aqueous solution with H_2_O_2_ as an oxidant.

Magnetite nanoflowers were synthesized in polyol solvents using *N*-methyl diethanolamine as a co-solvent through a non-classical crystallization route (Gallo-Cordova et al. 2022). The nanoflower material had a particle size of about 10 nm formed from the clustering of smaller Fe_3_O_4_ particles (3–4 nm in size). These multicore nanoparticles were applied for Fenton-like photocatalytic degradation of methyl orange under visible light and displayed high degradation reaction rates up to 8.8 10^−2^ min^−1^ (Gallo-Cordova et al. 2022).

In nano-composite structures, at least one of the phases of the combined materials has a nanoscale morphology. For example, a Fe_3_O_4_@SiO_2_@TiO_2_ nanocomposite was synthesized by the coprecipitation method and used for the photocatalytic degradation of methyl orange (Mazhari & Hamadanian 2018). The nanocomposite comprised a combination of three nanosized materials, viz., a nanoscale Fe_3_O_4_ core was coated with nanosized SiO_2_, which was again coated with nanosized TiO_2_ to form Fe_3_O_4_@SiO_2_@TiO_2_. The nanocomposite displayed high surface roughness, corresponding to a specific surface area of 257.67 m^2^/g and an average size of ~ 25 nm.

Nanohybrid material is not only described as a type of nanocomposite material but also contains an integration of organic and inorganic phases. The combination of two or more pre-synthesized nanomaterials could adapt the nanohybrids’ physicochemical properties and introduce new properties (Aich et al. 2014). A study by Xu et al. (2022) represented the synthesis of *Chlorella*@Fe_3_O_4_@BiOCl (CFB) microrobots for photocatalytic degradation of rhodamine B (RhB) dye. The CFB manufacturing process included the deposition of already prepared Fe_3_O_4_ onto *Chlorella* cells followed by the attachment of BiOCl nanosheets to the *Chlorella*@Fe_3_O_4_ surface. When applied for photocatalytic removal of RhB, The CFB biohybrid photocatalyst displayed a 95.6% removal efficiency in 30 min (Xu et al. 2022).

Single-phase magnetite-based nanoparticles are prepared from sole Fe_3_O_4_ without the combination of other materials. For example, a study by Kumar et al. (2016) synthesized sphere-shaped Fe_3_O_4_ nanoparticles using Andean blackberry leaf extract, where the particles revealed a cubic spinel phase and crystalline structure. When applied for dye-laden wastewater treatment, the nanoparticles displayed a methylene blue photocatalytic degradation rate of 0.0105475 min^−1^ under sunlight irradiation (Kumar et al. 2016).

## Synthesis and characterization techniques of magnetite-based photocatalysts

### Synthesis of magnetite-based photocatalysts

Nanomaterial synthesis techniques are selected to yield different shapes and compositions of magnetite-based photocatalysts. Various synthesis methods of photocatalysts and their synthesis procedures are given in Table 2.

**Table 2:** Synthesis techniques of magnetite-based photocatalysts

Photocatalyst	Synthesis technique	Technique description	Dye pollutant	Dye removal efficiency (%)	Ref
rGO-Fe_3_O_4_/TiO_2_ nanocomposite	Hydrothermal	rGO was dispersed in DI water, followed by stirring for 1 h. Fe_3_O_4_ solution was then added under vigorous stirring for 3 h. The sample was washed with DI water and dried at 110 °C for 12 h. rGO-Fe_3_O_4_ was then dispersed in DI water and sonicated for 1 h. Synthesized TiO_2_ was then dissolved in the as-prepared solution. The mixture was then poured into Teflon-lined stainless-steel autoclave and heated at 180 °C for 6 h. The sample was washed with DI water and dried at 110 °C for 12 h	Malachite green (MG) and methylene blue (MB)	99 and 97	Bibi et al. (2021)
Nanoflowers (α-MnO_2_-Fe_3_O_4_)	Hydrothermal	MnSO_4_ (0.25 mol/L) was dissolved in KMnO_4_ (0.4 mol/L) and vigorously stirred. Fe_3_O_4_ particles were added to the mixture, and then transferred to a Teflon-lined stainless-steel autoclave at 150 °C for 3 h. Afterwards, the system was allowed to cool naturally. The brown precipitate was washed with DI water and ethanol three times and dried at 60 °C overnight	Methylene blue (MB)	88	Rabani et al. (2022)
Fe_3_O_4_/TiO_2_/Ag Nanocomposites	Sol–gel method	TiO_2_ and NaOH were mixed in distilled water. This suspension was stirred at 80 °C. Fe_3_O_4_ and Ag nanoparticles were dispersed into a solution of ethanol and distilled water. After 2 h stirring and heating, the solution was collected, washed, aged, and then dried at 125 °C for 1 h under a vacuum	Methylene blue (MB)	85	Fauzian et al. (2017)
TiO_2_@Fe_3_O_4_ nanoparticles	Sol–gel method	HCl acid 37% was poured into distilled water, and pre-estimated fractions of FeCl_3_·6H_2_O and FeSO_4_·7H_2_O were added to the solution. Then, TiO_2_ nanoparticles were supplemented to the solution and placed on a stirrer. Nitrogen gas evolved, and NaOH was mixed with the solution inside the balloon and colour altered from brown to dark. The solution was filtered, and the washed precipitate was dried at 140 °C for 2 h	Direct Red 80 dye (DR-80)	100	Sadeghi et al. (2021)
Pomegranate-like Fe_3_O_4_/SiO_2_/TiO_2_ composite microspheres	Sol–gel	Fe_3_O_4_/SiO_2_ nanocomposites were dispersed in anhydrous ethanol. A certain amount of tetrabutyltitanate (TBOT) dissolved in isopropyl alcohol was added dropwise, followed by heating at 70 °C under vigorous stirring. After 12 h, the red-brown precipitates were washed with DI water and ethanol and kept at 60 °C for 8 h. Finally, and the products were calcined in air at 500 °C for 2 h	Methylene blue (MB)	78	Wang et al. (2012)
ZnO/Fe_3_O_4_-sepiolite nanoplatform	Sono-chemical	Fe_3_O_4_-SEP was dispersed in methanol, and the dispersion of ZnO in methanol was slowly added. The resulting dispersion was homogenized by ultrasound irradiation for 20 min with 10 kJ in sequential pulses (10 s on/off) at 50% of amplitude with a 13-mm tip. The resulting material was dried at 40 °C and ground	Methylene blue (MB)	100	Akkari et al. (2017)
Ternary magnetic ZnO/Fe_3_O_4_/g-C_3_N_4_ composite	Ultrasonication	A homogeneous mixture was obtained by vigorously grinding ZnO/Fe_3_O_4_ and melamine (mass ratio of 1:1) and then dispersing the mixture in DI. The suspension was ultrasonicated for 1 h. After that, the precursors were dried at 70 °C overnight, and the delivered solid was annealed at 550 °C for 2 h	Alizarin yellow R (AYR)	98.05	Wu et al. (2019)
Fe_3_O_4_@SiO_2_/MoO_3_/PDA-GO composites	Co-deposition	FSM material was dispersed in 10 mM Tris buffer solution (pH = 8.5) by sonication for 5 min. D-HCl and GO were added and sonicated with the as-prepared suspension for 5 min. Then, the mixture was stirred at 40 °C for 24 h. The resulting black composite was magnetically collected, washed with DI water until pH 7, and then freeze-dried	Methylene blue (MB)	98	Vasallo-Antonio et al. (2021)
Fe–p-C_3_N_4_	Pyrolysis method	Urea and FeCl_3_ were dissolved in distilled water (molar ratio urea/Fe = 0.12%). After solvent evaporation, the solid compound was heated in the muffle furnace. For the treatment with H_2_O_2_, synthesized Fe–p-C_3_N_4_ was mixed with H_2_O_2_ solution. An ice bath was used to control the temperature of the mixture and slow down the reaction. After 3 h, the suspension was centrifuged, and the solid was treated again with 10 mL H_2_O_2_ for an additional 3 h. Afterwards, the material was centrifuged and dried at 80 °C for 16 h	Methyl orange (MO)	70	Vu et al. (2020)
Fe_3_O_4_/SnO_2_ nanocomposites	Hydrothermal	Prepared Fe_3_O_4_ nanoparticles were dispersed in DI water by ultrasonication. SnCl_2_·2H_2_O and NaOH were mixed thoroughly in distilled water under stirring. Fe_3_O_4_ nanoparticles were mixed into the above solution, and the mixture was transferred into an autoclave. The hydrothermal reaction was maintained at 200 °C for 8 h. The final precipitate was washed and dried at 80 °C overnight	Crystal Violet Dye	83	Vinosel et al. (2019)
Zn_0.5_Fe_3-0.5_O_4_ nanoparticles	Solvothermal Reflux	Benzyl ether and oleylamine solvents mixture were considered as a reaction solvent. The mixture was stirred twice for 10 min (i.e., before and after supplementing stoichiometric metal precursor fine powders to the reaction solvent mixture). Oleic acid (10 mL) was added as a surfactant. The resultant reactant mixture was heated to the boiling point of the solvent’s mixture (300 °C) at a heating rate of 5 °C/min for 1 h. The reaction mixture colour turned black, confirming the nanoparticles (ferrite) generation, and then cooled to room temperature. Finally, ethanol was supplemented to the reaction mixture for purification, and the nanoparticles were separated by centrifugation	Rhodamine blue (RhB)	97.31	Manohar et al. (2021)
*Chlorella*@Fe_3_O_4_@BiOCl microrobots (CFB)	Co-precipitation	As-prepared magnetic composites and Bi(NO_3_)_3_⋅5H_2_O were mixed in DI water and ultrasonicated for 30 min. Acetic acid was added under vigorous stirring. The brown suspension was stirred for 30 min, and then an aqueous solution containing NaCl and NaAc was supplemented. After an extra 2 h of stirring and 1 h of aging, the resulting precipitate was collected and washed three times. Finally, the synthesized CFB microrobots were freeze-dried	Rhodamine B (RhB)	95.6	Xu et al. (2022)
Fe_3_O_4_@TiO_2_@PDA/SiW11V-Ag multicomponent composite	Solvothermal and Co-precipitation	Fe_3_O_4_ magnetite particles were synthesized via a facile one-step solvothermal pathway using a partial reduction co-precipitation method. TiO_2_ shell was constructed by hydrolysis of tetrabutyl titanate precursor in the suspension of Fe_3_O_4_ magnetite particles and further treated at 500 °C under N_2_ atmosphere. Mono-vanadium substituted silicotungstate (SiW_11_V) decoration was maintained via polymerizations of dopamine in alkaline conditions (pH = 8.5). After UV irradiation for 2 h, Ag nanoparticles are deposited on the Fe_3_O_4_@TiO_2_@PDA/SiW_11_V surface in propan-2-ol medium and N_2_ condition	Methyl orange (MO)	100	Wu et al. (2021)
MOF-1/GO/Fe_3_O_4_ nanocomposite	Hydrothermal	A mixture of NdCl_3_·6H_2_O, H_3_TCPB, distilled water, C_2_H_5_OH, and N,N-dimethylformamide (DMF) were stirred for 30 min and transferred into a stainless steel autoclave. After a hydrothermal process under autogenous pressure at 393 K for 72 h, the reactor was naturally cooled. The purple bulk crystals were collected by filtration, washed with H_2_O, and dried in air	Methylene blue (MB)	95	Bai et al. (2020)
Magnetic BiOBr/Fe_3_O_4_/RGO composites	Hydrothermal	BiOBr was added to ethylene glycol, and the solution was stirred to completely disperse. Fe_3_O_4_/RGO was slowly added to the as-prepared mixture and stirred for 4 h. The hydrothermal reaction was carried out at 160 °C for 12 h using a Teflon stainless steel reactor connected to polytetrafluoroethylene. The final product was washed with water and ethanol several times, and dried under vacuum for 12 h to obtain a powdery sample	Rhodamine B (RhB)	96	Zheng et al. (2020)
Fe_3_O_4_/ZnO/Si_3_N_4_ nanocomposite	Reduction Method	Synthesized Fe_3_O_4_/ZnO bimetal oxide was properly mixed with silica and urea. Under a N_2_ condition, the mixture was heated at a ramping rate of 10 °C/min to obtain 600 °C for 2 h	Methyl orange (MO)	96	Sharma et al. (2020)
TiO_2_/Fe_3_O_4_	Modified co-precipitation	Solution “A” included TiO_2_ in ethanol. Solution “B” comprised Fe_3_O_4_ in DI water. Both solutions were individually ultrasonicated for 30 min to get uniform dispersions. Subsequently, solution “B” was added dropwise to solution “A” under ultrasonication until the formation of a uniform dispersion The final precipitate was washed with DI water and dried at 70 °C overnight	Orange G	60	Mercyrani et al. (2018)

The hydrothermal method, sol–gel synthesis technique, and ultrasonication process have been commonly used to prepare magnetite-based photocatalysts.

The hydrothermal synthesis technique involves the use of aqueous media as a reaction system, where wastewater is pressurized and heated to supercritical conditions in a reactor vessel (Bibi et al. 2021). This solution reaction–based approach is properly used to prepare nanomaterials whose precursors are insoluble at room temperature and pressure. It can control the shape, size, particle distribution, and alignment of the synthesized nanomaterials through varying temperatures and pressures.

A two-step hydrothermal method was used to synthesize α-MnO_2_-Fe_3_O_4_ three-dimensional nanoflower-like structure for the treatment of wastewater containing dyes (Rabani et al. 2022). The synthesis process involved the dispersion of Fe_3_O_4_ nanoparticles into a solution of potassium permanganate, and the mixture was allowed to stir vigorously. The solution was then transferred to a Teflon-lined stainless-steel autoclave and retained at 150 °C for 3 h and then allowed to cool gradually at room temperature. The nanoflowers were then accumulated as a brown precipitate, which was then washed and dried at 60 °C overnight. When applied for photocatalytic experiments, the nanoflowers displayed high degradation of methylene blue and crystal violet dyes of 94.8% and 93.7%, respectively.

The sol–gel process of nanomaterial preparation involves the use of a solution of the desired nanomaterial precursor called a sol. The sol is then subjected to hydrolysis or condensation, causing an increase in viscosity and converting the solution to the “gel” form (Patil et al. 2021). The gel is further separated into liquid and solid phases by filtration, centrifugation, or sedimentation. Once separated, the solid part is dried to remove the moisture to finally yield the desired nanomaterial (Saeed et al. 2019). The sol–gel method is also simple, economical, and efficient, producing high-quality nanomaterials (Modan and Schiopu 2020). Magnetite–based photocatalysts were successfully synthesized using the sol–gel method and utilized for textile wastewater treatment, depicting dye photocatalytic degradation efficiencies of > 80% in each study (Fauzian et al. 2017; Sadeghi et al. 2021; Wang et al. 2012).

Nanocomposites of Fe_3_O_4_/TiO_2_/Ag were synthesized by suspending TiO_2_ nanoparticles in a solution of sodium hydroxide followed by stirring at 80 °C (Fauzian et al. 2017). A mixture of Fe_3_O_4_ and silver nanoparticles dispersed in a solution of ethanol and distilled water was then added to the suspension and allowed to mix continuously under heating for 2 h. The mixture was then aged to form the gel and then dried at 125 °C for 1 h to obtain the nanocomposite. Applying this nanocomposite for photocatalytic degradation of methylene blue dye gave a high degradation efficiency of 85% within 120 min.

Ultrasonication is also one of the commonly applied nano-photocatalyst synthesis techniques. This method involves the exposure of nanomaterial precursor solutions to ultrasonic irradiation, forming ultrasonic cavitation of the solution (Rane et al. 2018). These developed cavities attempt to continuously absorb and concentrate the diffused sound energy. Sequentially, the cavity bubbles exhibit rapid growth and can no longer absorb the energy efficiently. The liquid will rush into the cavities, leading to cavity implosion. As a result, energy is released to change the solution temperature and pressure, preventing the agglomeration and growth of nanoparticles into larger sizes.

The ultrasonication technique was employed to synthesize a ternary magnetic ZnO/Fe_3_O_4_/g-C_3_N_4_ composite (Wu et al. 2019). As such, ZnO/Fe_3_O_4_ and melamine were ground and dispersed into deionized water, and the formed mixture (with a mass ratio of 1:1) was ultrasonicated for 1 h. The obtained mixture was then dried at 70 °C overnight to remove the solvent and then annealed at 550 °C for 2 h to obtain the nanocomposite. The nanocomposite was applied for treating mono azo dyes-rich wastewater, achieving photocatalytic degradation efficiencies of 97.87%, 83.35%, and 98.05% for methyl orange, orange G, and alizarin yellow R, respectively.

### Characterization of magnetite-based photocatalysts

Different types of dyes have been subjected to the photocatalytic degradation process. The behaviour of magnetite-based nanomaterials for photocatalysis could be described using the physical/mechanical, chemical, optical, and electrical parameters. These parameters are determined using the nanomaterial characterization by X-ray diffraction analysis (XRD), Fourier transform infrared spectroscopy (FTIR), scanning electron microscopy (SEM), and vibrating sample magnetometer (VSM) (Table 3).

**Table 3:** Magnetite-based photocatalysts characterization methods

Photocatalyst	Characteristic peaks relating to Fe_3_O_4_ observed	Ref
rGO-Fe_3_O_4_ /TiO_2_ nanocomposite	XRD: rGO was confirmed by 2*θ* = 25.4° corresponding to (0 0 2) plane FTIR peaks at 1616, 1730, 1394, and 1219 cm^−1^ were related to rGO; peaks around 500–560 cm^−1^ were related to Ti–O and Ti–O-Ti; band at 581 cm^−1^ corresponds to Fe–O SEM shows that TiO_2_ surrounded the Fe_3_O_4_ nanoparticles anchored on rGO sheets EDS showed Ti, O, and Fe elements	Bibi et al. (2021)
Manganese dioxide-incorporated iron oxide three-dimensional nanoflowers (α-MnO_2_-Fe_3_O_4_)	XRD complied with the standard (JCPDS No. 19–0629 and 44–0141) for Fe_3_O_4_ and α-MnO_2_ α-MnO_2_-Fe_3_O_4_ 3D-flower-like spherical structure with 82 m^2^/g, respectively	Rabani et al. (2022)
Fe_3_O_4_/TiO_2_/Ag Nanocomposites	XRD: TiO_2_ structure was detected at 2*θ* = 25.2°, and 37.8° for (100) and (004) planes, respectively; cubic structure from Ag was found at 2*θ* = 37.52°, 44.61°, and 64.57°	Fauzian et al. (2017)
Titanium dioxide nanoparticles on the surface of magnetite nanoparticles (TiO_2_@Fe_3_O_4_)	XRD: 2*θ* = 30.15°, 35.5°, 43.13°, 53.49°, 56.99°, 62.57°, and 70.97° for Fe_3_O_4_ FTIR = 520–740 cm^−1^ corresponding to tensile stretching of Ti–O and Fe–O	Sadeghi et al. (2021)
ZnO/Fe_3_O_4_-sepiolite nanoplatform (ZnO/Fe_3_O_4_-SEP)	XRD: 2*θ* = 30.4°, 35.6°, 43.3°, 53.6°, 57.3°, and 62.7° for Fe_3_O_4_ 2*θ* = 31.9°, 34.2°, and 36.3° for ZnO hexagonal wurtzite lattice SEM shows ZnO nanoparticles covering the sepiolite fibres Specific surface area (*S*_BET_) = 114 m^2^/g	Akkari et al. (2017)
Ternary magnetic ZnO/Fe_3_O_4_/g-C_3_N_4_ composite	XRD: 2*θ* = 31.81°, 34.44°, 36.21°, 47.60°, 56.62°, 63.01°, and 67.97° for the hexagonal wurtzite structure of ZnO; 2*θ* = 27.3° for g-C_3_N_4_; 2*θ* = 30.4°, 35.7°, and 43.4° for Fe_3_O_4_ The sample was superparamagnetic The sample had layered platelet-like morphology structure	Wu et al. (2019)
Fe_3_O_4_@SiO_2_/MoO_3_/PDA-GO composites	XRD showed the cubic spinel structure of Fe_3_O_4_ (JCPDS 19–0629); GO depicted a strong (001) peak at 2*θ* = 12° FTIR: 564 cm^−1^ due to the Fe–O stretching of the Fe_3_O_4_ core	Vasallo-Antonio et al. (2021)
Fe–p-C_3_N_4_	XRD: 2*θ* = 35.5° for the (311) crystal plane of Fe_3_O_4_ Absorption bands at 1200–1600 cm^−1^ for stretching mode of typical C–N heterocycles SEM showed porous stacked layers as the typical morphology of p-C_3_N_4_ SSA = 69 m^2^/g; pore volume = 0.3 cm^3^/g; band gap = 2.8 eV	Vu et al. (2020)
Fe_3_O_4_/SnO_2_ nanocomposites	XRD: 2*θ* = 30.2°, 35.5°, 43.1°, 57.2°, and 62.5° for cubic inverse spinel structure of Fe3O4 2*θ* = 26.6°, 33.9°, 37.8°, 51.9°, 54.8°, and 61.8° for tetragonal rutile crystalline phase SnO_2_ FTIR: absorption band at 584 cm^−1^ denotes Fe–O stretching vibrations of Fe_3_O_4_ A prominent band at 593 cm^−1^ represents a characteristic peak of Sn–O stretching vibrations of SnO_2_ nanoplates Absorption peak in 490–590 cm^−1^ shows the combination of Fe–O and Sn–O EDAX spectra reveal the presence of Fe, Sn, and O elements	Vinosel et al. (2019)
Zn_0.5_Fe_3-0.5_O_4_ NPs	XRD: 2*θ* = 29.9°, 35.3°, 42.9°, 53.2°, 56.7°, 62.3°, and 74.7° for the cubic inverse spinel structure ZnFe_2_O_4_ (ICDD No. 89–7412) FTIR: peaks < 1000 cm^−1^ for the Fe–O bands Magnetic field frequency = 316 kHz and amplitude = 35.28 kAm^−1^	Manohar et al. (2021)
Fe_3_O_4_@TiO_2_@PDA/SiW11V-Ag multicomponent composite	XRD: 2*θ* = 30.0°, 35.3°, 42.9°, 53.5°, 57.0°, and 62.4° for cubic phase of Fe_3_O_4_; 2*θ* = 25.3°, 37.9°, 48.0°, and 53.9° for anatase phase of TiO_2_ shell; 2*θ* = 37.9°, 44.1°, 64.3°, and 77.2° for face-centred-cubic structure of Ag FTIR: band at 584 cm^−1^ for stretching vibration of Fe − O bonds; 954 cm − 1 for Ti − O − Ti stretching vibrations	Wu et al. (2021)
MOF-1/GO/Fe_3_O_4_ nanocomposite	XRD: 2*θ* = 11.14° for GO FTIR: 588 cm^−1^ for Fe–O vibration BET surface area = 48.80 m^2^/g A large number of Fe_3_O_4_ microspheres were uniformly loaded on GO surface	Bai et al. (2020)
Magnetic BiOBr/Fe_3_O_4_/RGO composites	XRD patterns of cubic Fe_3_O_4_ nanoparticles (JCPDS#65–3170) FTIR: a peak at 588 cm^−1^ for Fe–O vibration Pure BiOBr with band gap energy of 2.58 eV	Zheng et al. (2020)
TiO_2_/Fe_3_O_4_	XRD: 2*θ* = 30.14°, 35.50°, 43.15°, 57.07°, and 62.67° for inverse cubic spinel structure of Fe_3_O_4_ (ICDD card no.71–6336); 2*θ* = 25.28°, 37.80°, 48.04°, 53.88°, 55.06°, 62.68°, 68.76°, 70.30°, and 75.02° for tetragonal structure of TiO_2_ (anatase-ICDD card no. 21–1272) SEM displays large pieces, chunks of several µm with finer particles, and agglomerates of various sizes	Bastos-Arrieta et al. (2018)

The crystalline structure, phase nature, lattice parameters, and crystalline grain size of the fabricated photocatalyst are measured by XRD (Mourdikoudis et al. 2018). Magnetite possesses an inverse spinel cubic structure and conforms to the F3dm JCPDS card (Abdel Maksoud et al. 2020). When a beam of X-rays is directed toward the sample and contacts a crystalline substance/phase, it gets scattered by atoms in the sample (Chhantyal 2022; Sharma et al. 2012). The interference patterns are detected (Chhantyal 2022), and the generated peak characteristics (position and intensity) are compared with the reference patterns available from the International Centre for Diffraction Data (ICDD, previously known as the Joint Committee on Powder Diffraction Standards, JCPDS) database (Mourdikoudis et al. 2018). XRD characterization was performed on the synthesized magnetite-based photocatalysts before and after their application for dyes’ photodegradation (Mazhari et al. 2018; Zhang et al. 2013). In a prepared Fe_3_O_4_@SiO_2_@AgCl/Ag/Ag_2_S magnetic photocatalyst (Mazhari et al. 2018; Zhang et al. 2013), the diffraction peaks of Fe_3_O_4_, AgCl, and Ag_2_S were assigned by JCPDS, no. 75–0449, JCPDS file: 85–1355, and JCPDS file: 89–3840, respectively. The XRD patterns of Fe_3_O_4_@MIL-100(Fe) microspheres complied with the cubic phase Fe_3_O_4_ (JCPDS card, file No. 89–4319) and other peaks for the crystalline MIL-100(Fe) (Guidolin et al. 2021).

FTIR provides information about the photocatalyst’s surface molecular composition (Mourdikoudis et al. 2018; Eid 2022). This analysis is carried out by the irradiation of electromagnetic radiation, having wavelengths in the mid-infrared region (4000–400 cm^−1^), onto the material (Sindhu et al. 2015). The material’s molecules become infrared active once they absorb the radiation, generating an FTIR spectrum (Mourdikoudis et al. 2018). This spectrum displays absorption peaks equivalent to the vibration frequencies of atoms inside the photocatalyst. FTIR has been used as a characterization technique on synthesized magnetite-based nanomaterials (rGO-Fe_3_O_4_/TiO_2_ nanocomposite, TiO_2_@Fe_3_O_4_, ZnO/Fe_3_O_4_-SEP, Fe_3_O_4_@SiO_2_/MoO_3_/PDA-GO composite, Fe_3_O_4_/SnO_2_, Fe_3_O_4_@TiO_2_@PDA/SiW11V-Ag, MOF-1/GO/Fe_3_O_4_ nanocomposite, and magnetic BiOBr/Fe_3_O_4_/RGO composite), as previously reported (Akkari et al. 2017; Bai et al. 2020; Bibi et al. 2021; Sadeghi et al. 2021; Vasallo-Antonio et al. 2021; Vinosel et al. 2019; Wu et al. 2021; Zheng et al. 2020). For instance, the FTIR spectrum of the synthesized Fe_3_O_4_/SiO_2_/TiO_2_ composites showed peaks at 800 cm^−1^ and 1080 cm^−1^ for Si–O–Si, 940–960 cm^−1^ for Si–O–Ti, and 500–900 cm^−1^ for Ti–O–Ti (Wang et al. 2012).

SEM is another technique used to characterize magnetite-based photocatalysts by yielding direct imaging and measurements of the material morphology (Torres-Rivero et al. 2021). SEM also gives information about the material chemical composition (Bastos-Arrieta et al. 2018; Zhou et al. 2006). This technique works by directing a beam of high-energy electrons towards the material to be characterized (Zhou et al. 2006). Once the beam interacts with the material, the material’s electrons are excited and scattered (Zhou et al. 2006). The scattered electrons are received/collected by an Everhart Thornly (ET) detector, which amplifies the electron energies to generate characterization images. The SEM image was used to investigate the microstructure and morphology of a synthesized BiOBr/Fe_3_O_4_/RGO composite utilized for rhodamine B dye photodegradation (Zheng et al. 2020). The SEM examination showed that the photocatalytic activity of the composite material was enhanced by the strong connection between the Fe_3_O_4_/RGO sheets and BiOBr.

VSM is another characterization method and is used to measure the magnetic moment centres in photocatalysts, following Faraday’s law of induction (Adeyeye and Shimon 2015). The magnetic property is measured by vibrating the material within a constant/uniform magnetic field (often between 50 and 100 Hz) to generate an electric current in suitably positioned sensing coils (Thomson 2014). The sample is connected to a vibrating head module and positioned between two pickup coils (electromagnet poles). The magnetic behaviour of the sample includes saturation magnetization, coercivity, switching fields, and remnant magnetization. The magnetic moment information varies according to the nanoparticles’ morphology, synthetic conditions, shape, and size (Adeyeye and Shimon 2015).

## Factors influencing dye removal by magnetite-based photocatalysts

Several operating factors, including substrate pH, dye concentration, and catalyst dosage, influence the efficiency of magnetite-based photocatalysts in degrading textile dyes:

### Effect of pH

The solution pH has a great impact on the adsorption properties of the photocatalyst. More positive charges tend to accumulate on the photocatalyst surface when the substrate pH is below the photocatalyst’s pH for the zero-charge point (pH_ZPC_), providing a suitable condition for anionic dye attraction. At pH > pH_ZPC_, the photocatalyst surface gets a net negative charge and attracts the cationic (positively charged) dyes (Alkaim et al. 2014). The pH_ZPC_ value can be derived from the pH drift method where the same mass of catalyst solution is suspended in electrolyte solutions of different pH values and a plot of the change in pH (ΔpH) *vs*. initial pH is generated, revealing the pH_ZPC_ as the intersection point. The pH_ZPC_ value can also be obtained from the mass titration method, where a concentrated solution of the catalyst material is made by suspending the catalyst in distilled water and the pH_ZPC_ is taken as the natural pH of the dispersion; the pH_ZPC_ is taken as the intersection point of a graph of inert electrolyte against single titration (Kosmulski 2023). For example, the pH_ZPC_ of TiO_2_ was 6.5, giving the highest photocatalytic efficiency at 7 pH for malachite green and 9 pH for methylene blue (Bibi et al. 2021). As such, the interaction of dyes with the net charge on the photocatalyst surface is also governed by the dye type (either cationic dyes or anionic dyes). Moreover, a highly acidic condition could result in leaching Fe^2+^ and Fe^3+^ ions from the magnetite photocatalyst surface into the solution (Abdelhaleem et al. 2019; Guidolin et al. 2021). These leached ions tend to react with a hydrogen peroxide (H_2_O_2_) oxidant, following a Fenton-like reaction (Eqs. 9 and 10).

9 $${{\text{Fe}}}^{2+}+ {{\text{H}}}_{2}{{\text{O}}}_{2} \to {{{\text{Fe}}}^{3+}+{\text{OH}}}^{-}{+}^{\bullet }{\text{OH}}$$

10 $${{\text{Fe}}}^{3+}+ {{\text{H}}}_{2}{{\text{O}}}_{2} \to {{{\text{Fe}}}^{2+}+{\text{H}}}^{+}+{{\text{OOH}}}^{\bullet }$$

### Effect of photocatalysis time

The photocatalytic degradation of dyes in aqueous solution is generally improved by prolonging the reaction time. The photogenerated reactive oxygen species (ROS) and charge carriers get trapped onto the photocatalyst surface and then interact with the dye molecules. The effect of time on dyes’ photodegradation also corresponds to the saturation state of the catalyst surface with dye molecules. Photocatalytic degradation would continue to increase until the catalyst surface becomes saturated, i.e., an approximately horizontal line in a degradation *vs*. time plot represents a stabilization in the photocatalytic degradation rate.

### Effect of catalyst dosage

**Fig. 3:**
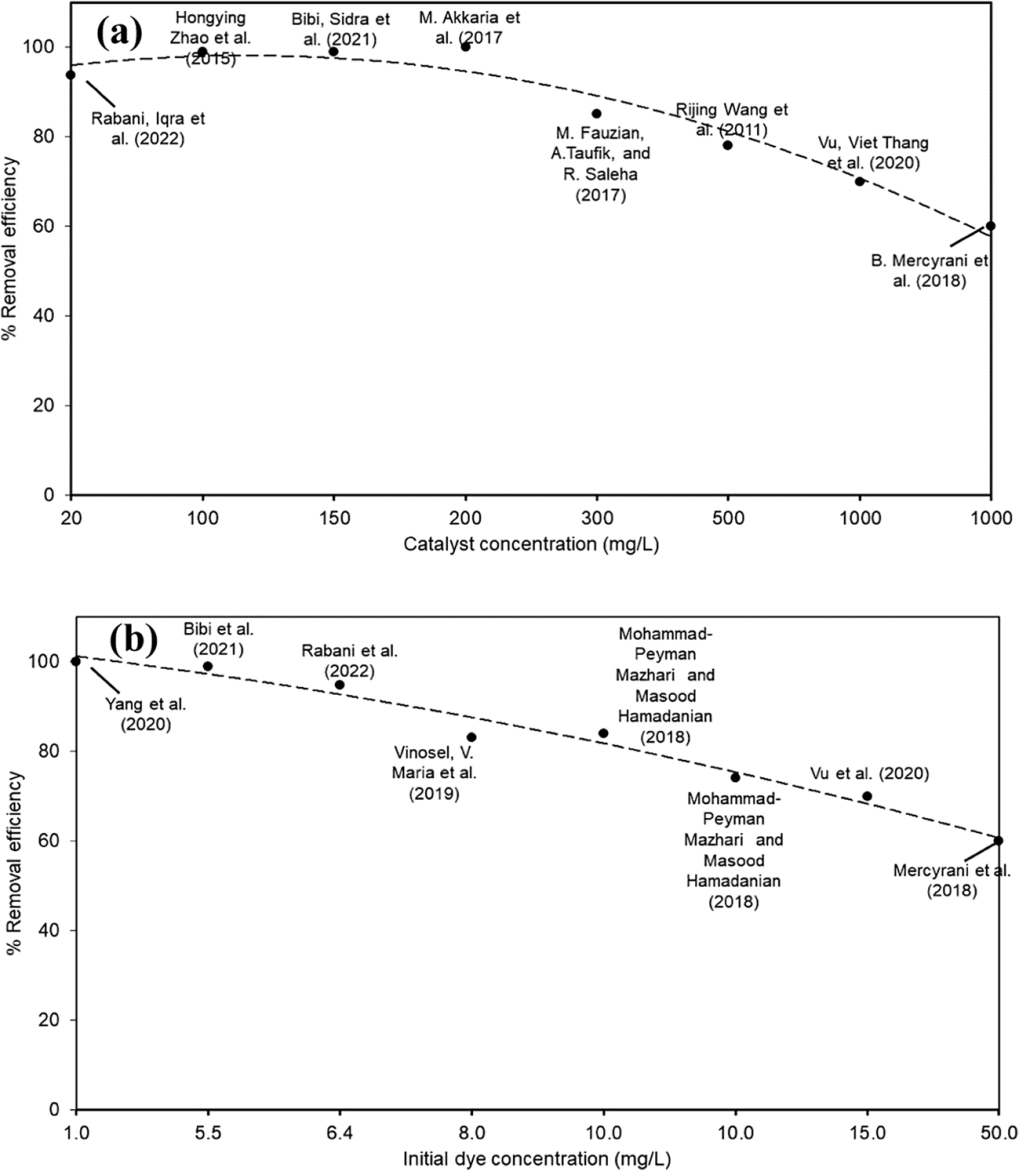
Graphs of dye removal against (**a**) catalyst dose and (**b**) initial dye concentration obtained from bibliometric data

Figure 3a shows the effect of increasing the photocatalyst dosage on dye removal using data reported in the literature.

The degradation efficiencies are enhanced by increasing the photocatalyst dosage due to the availability of abundant active sites (Abdelhaleem and Chu 2018), increasing the number of free radicals. However, this degradation performance begins to decline after increasing the photocatalyst dosage beyond the optimum value. This pattern was observed in Vasallo-Antonio et al. (2021), where methylene blue degradation of 98.0% was observed at 200 mg/L Fe_3_O_4_@SiO_2_-MoO_3_-polydopamine-graphene oxide composite photocatalyst under illumination within 60 min. As the catalyst is overdosed, the solution turbidity increases and the light penetration into the solution becomes deficient. This pattern occurs due to reflecting a portion of light from the medium, which reduces the efficiency of hydroxyl radical generation. Increasing the ZnO/biosilica nanocomposite within the 0.1–1.5-g/L range enhanced the decolorization of Acid Red 88 (AR88) from 15.30 to 98.54%, respectively, while this efficiency dropped to 93.63% at a dosage of 2 g/L (Darvishi Cheshmeh Soltani et al. 2015). Increasing the photocatalyst dosage might also be accompanied by particles’ agglomeration, causing loss of the specific surface area and the number of activated sites (Gnanaprakasam et al. 2015).

### Effect of initial dye concentration

Figure 3 b reveals a reduction in the photocatalytic degradation efficiencies with increasing the initial dye concentrations (*C*_*o*_), probably due to the inefficient adsorption of O_2_ and OH^−^ on the photocatalyst. At higher *C*_*o*_, most of the active sites on the photocatalyst become occupied and unable to receive additional dye molecules (Darvishi Cheshmeh Soltani et al. 2015; Salama et al. 2018). Moreover, this condition permits the absorption of a higher percentage of light irradiance on the dye molecules instead of the photocatalyst particles, thereby reducing the catalyst’s potential for ROS generation. In a photodegradation process by composite nanofibers, elevating *C*_*o*_ from 10 to 50 ppm reduces the removal efficiencies of different dye compounds from ~ 100 to 70%, respectively (Darvishi Cheshmeh Soltani et al. 2015; Salama et al. 2018). Decreasing the dye degradation efficiency at higher *C*_*o*_ was also noticed due to the shortening of the photon path length entering the dye solution (i.e., photons could be blocked before reaching the photocatalyst surface), and the competition between the generated intermediates and dye molecules for the adsorption and photocatalytic sites.

### Effect of interfering ions and humic acid

Dye-laden wastewaters usually contain mineral ions, such as Fe^2+^, Zn^2+^, Ag^+^, Na^+^, Cl^−^, PO_4_^3−^, SO_4_^2−^, BrO_3_^−^, CO_3_^2−^, HCO_3_^−^, and S_2_O_8_^2−^ (Biswas et al. 2022; Rauf and Ashraf 2009), originated from the additives used to improve the textile manufacturing processes. For instance, the CO_3_^2−^ and HCO_3_^−^ ions are usually added to the dye bath in textile industries for pH adjustment. These ions, based on their concentrations in the medium, attempt to compete with dye molecules for the photocatalytic active sites (Chong et al. 2010). Moreover, some ions, such as Fe^2+^, CO_3_^2−^, HCO_3_^−^, Cl^−^, and SO_4_^2−^, react with OH^•^ radicals (Eqs. 11–13), generating much less reactive ROS (Muruganandham and Swaminathan 2006; Nguyen et al. 2020; Rauf and Ashraf 2009; Rauf et al. 2007). These reactions decrease the HO^•^ radical concentrations, retarding the photocatalysis rate for dye degradation:

11 $${{\text{Fe}}}^{2+}{+}^{\bullet }\mathrm{OH }\to {{{\text{Fe}}}^{3+}+{\text{OH}}}^{-}$$

12 $${{{\text{CO}}}_{3}}^{2-}+ \dot{}{\text{OH}}\to {{{{\text{CO}}}_{3}}^{\bullet -}+{\text{OH}}}^{-}\,k = 3.9 \times 108$$

13 $${{{\text{HCO}}}_{3}}^{-}+ \dot{}{\text{OH}}\to {{{\text{CO}}}_{3}}^{\bullet -}+{{\text{H}}}_{2}{\text{O}}\,k = 8.5 \times 106$$

The presence of Cl^−^ in the dye solution contributes to hole and hydroxyl radical scavenging effect; i.e., Cl^−^ could react with the photogenerated holes and hydroxyl radicals (Abdelhaleem et al. 2020), where their scavenging effects could be derived in Eqs. 14–17:

14 $${{\text{Cl}}}^{-}+{h}_{{\text{VB}}}^{+}{\to }^{\bullet }{\text{Cl}}$$

15 $${{\text{Cl}}}^{-}{+}^{\bullet }{\text{Cl}}\to {{\text{Cl}}}^{\bullet -}$$

16 $${{\text{CI}}}^{-}{+}^{\bullet }{\text{OH}}\to {{\text{HOCI}}}^{\bullet -}$$

17 $${{\text{HOCI}}}^{\bullet -}+{{\text{H}}}^{+}{\text{CI}}+{{\text{H}}}_{2}{\text{O}}$$

The reactions of sulphate ions with the photogenerated holes and ^•^OH radicals in dye-contaminated solution are given by Eqs. 18 and 19 (Abdelhaleem and Chu 2019):

18 $${{{\text{SO}}}_{4}}^{2-}+{h}_{{\text{VB}}}^{+}\to {{\text{SO}}}_{4}^{\bullet -}$$

19 $${{{\text{SO}}}_{4}}^{2-}{+}^{\bullet }{\text{OH}}\to {{\text{SO}}}_{4}^{\bullet -}+{{\text{OH}}}^{-}$$

Simultaneously, the ions released in wastewaters may also decline the photocatalytic activity by scavenging electrons, resulting in reduced O_2_^•–^ radical formation (Rauf et al. 2007).

Persulphate ion (S_2_O_8_^2−^) is a strong oxidizing agent that can release sulphate ions in solution, reducing the entire photocatalytic performance (Eq. 20):

20 $${{{\text{S}}}_{2}{{\text{O}}}_{8}}^{2-}+{{e}_{{\text{cb}}}}^{-}\to {{{\text{SO}}}_{4}}^{\bullet -}+{{{\text{SO}}}_{4}}^{2-}$$

The sulphate ions can then react with ^•^OH radicals, as represented in Eq. 19. The sulphate radicals can further react with water molecules to produce more sulphate ions as follows (Eq. 21):

21 $${{{\text{SO}}}_{4}}^{\bullet -}+{{\text{H}}}_{2}{\text{O}}{\to }^{\bullet }{\text{OH}}+{{{\text{SO}}}_{4}}^{2-}+{{\text{H}}}^{+}$$

Because SO_4_^2−^ is less reactive than ^•^OH radicals, SO_4_^2−^ accumulation in the medium might occur. This pattern reduces the dye photocatalytic degradation process.

Bromate ion (BrO_3_^−^) is an efficient electron scavenger. Therefore, it can react with conduction band electrons *e*^−^(CB) in the solution as follows (Eq. 22):

22 $${{\text{BrO}}}_{3}^{-}+6{e}^{-}\left({\text{CB}}\right)+6{{\text{H}}}^{+}\to {{\text{Br}}}^{-}+3{{\text{H}}}_{2}{\text{O}}$$

The bromide ions produced in the reaction can interact with ^•^OH radicals (Eq. 23), scavenging them and reducing their concentration in solution (Abdelhaleem et al. 2020; Rauf and Ashraf 2009):

23 $${{\text{Br}}}^{-}{+}^{\bullet }{\text{OH}}{\to }^{\bullet }{\text{Br}}+{{\text{OH}}}^{-}$$

Dye-laden wastewater (with textiles, paints, and varnishes) may also contain humic acids, which can bind to the nanoparticles’ surface (Carlos et al. 2011; Chandran et al. 2014). This binding not only reduces the nanoparticles’ adhesion and agglomeration but also negatively influences the photocatalyst performance. Chandran et al. (2014) showed that humic acid reduced the photocatalytic efficiency of the ZnO nanoparticles (as a photocatalyst), where the rate of MB photodegradation decreased at higher humic acid dosages. A humic acid concentration of 1 mg/L caused blockage to most of the TiO_2_ active site, reducing the tetracycline photocatalytic removal efficiency (Li and Hu 2016). This insufficient removal pattern could also be assigned to the quenching of ROS (^•^OH) with humic acid addition.

## Degradation and mineralization

The dye photodegradation mechanisms depend mainly on the photocatalyst and ROS types. For instance, the scavenging tests are performed by adding different ROS to the reaction chamber, followed by monitoring the shift in the photocatalytic removal efficiency (Fig. 1).

**Fig. 4:**
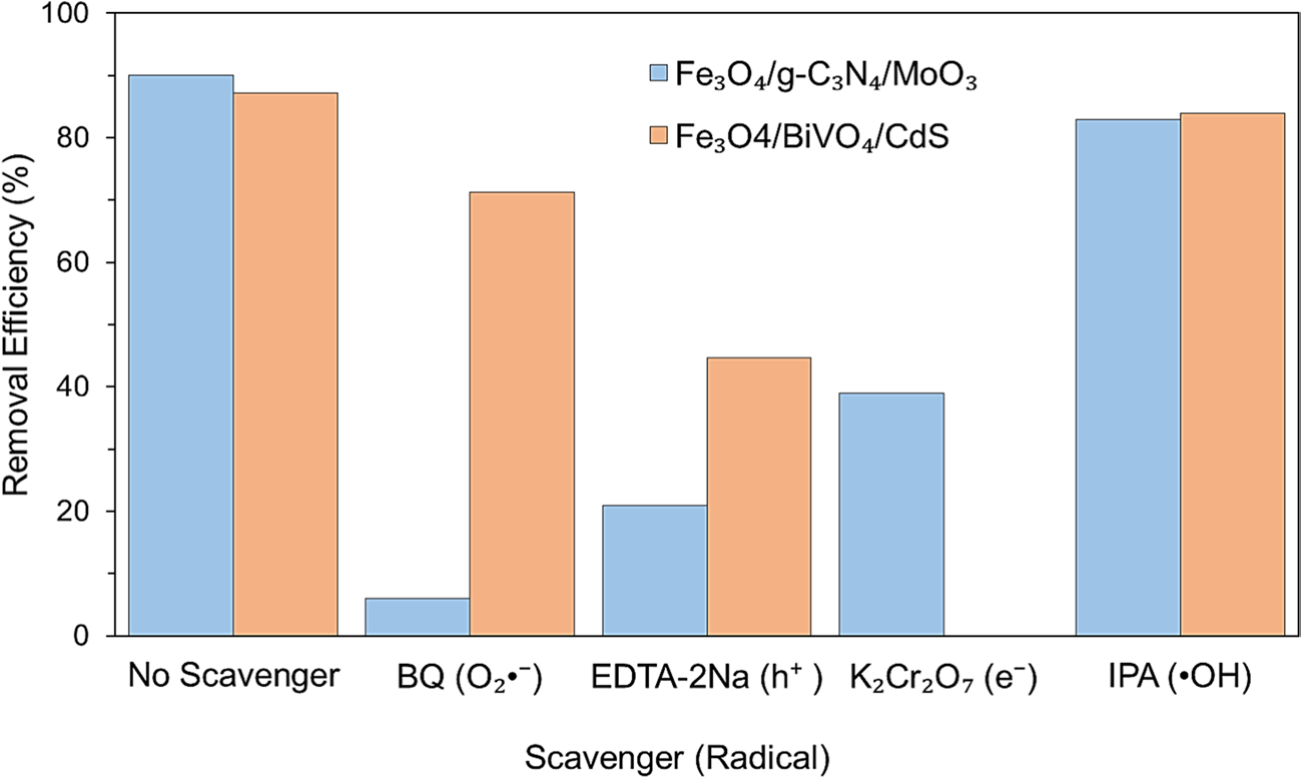
Effect of radical scavengers on dye photodegradation

Benzoquinone (BQ), potassium dichromate (K_2_Cr_2_O_7_), isopropanol (IPA), and ethylenediaminetetraacetate (EDTA-2Na) were employed as the quenchers (trapping agent) of O_2_^•−^, *e*^−^, ^•^OH, and *h*^+^, demonstrating that O_2_^•−^ was the major ROS for tetracycline degradation by magnetic nanocomposites (Fe_3_O_4_/g-C_3_N_4_/MoO_3_) (He et al. 2020). The scavenging test showed a drastic degradation efficiency from 90 to 6% in 120 min after BQ supplementation, whereas adding EDTA-2Na, K_2_Cr_2_O_7_, and IPA resulted in efficiency reductions from 90 to 21%, 39%, and 83%, respectively (Fig. 4).

**Fig. 5:**
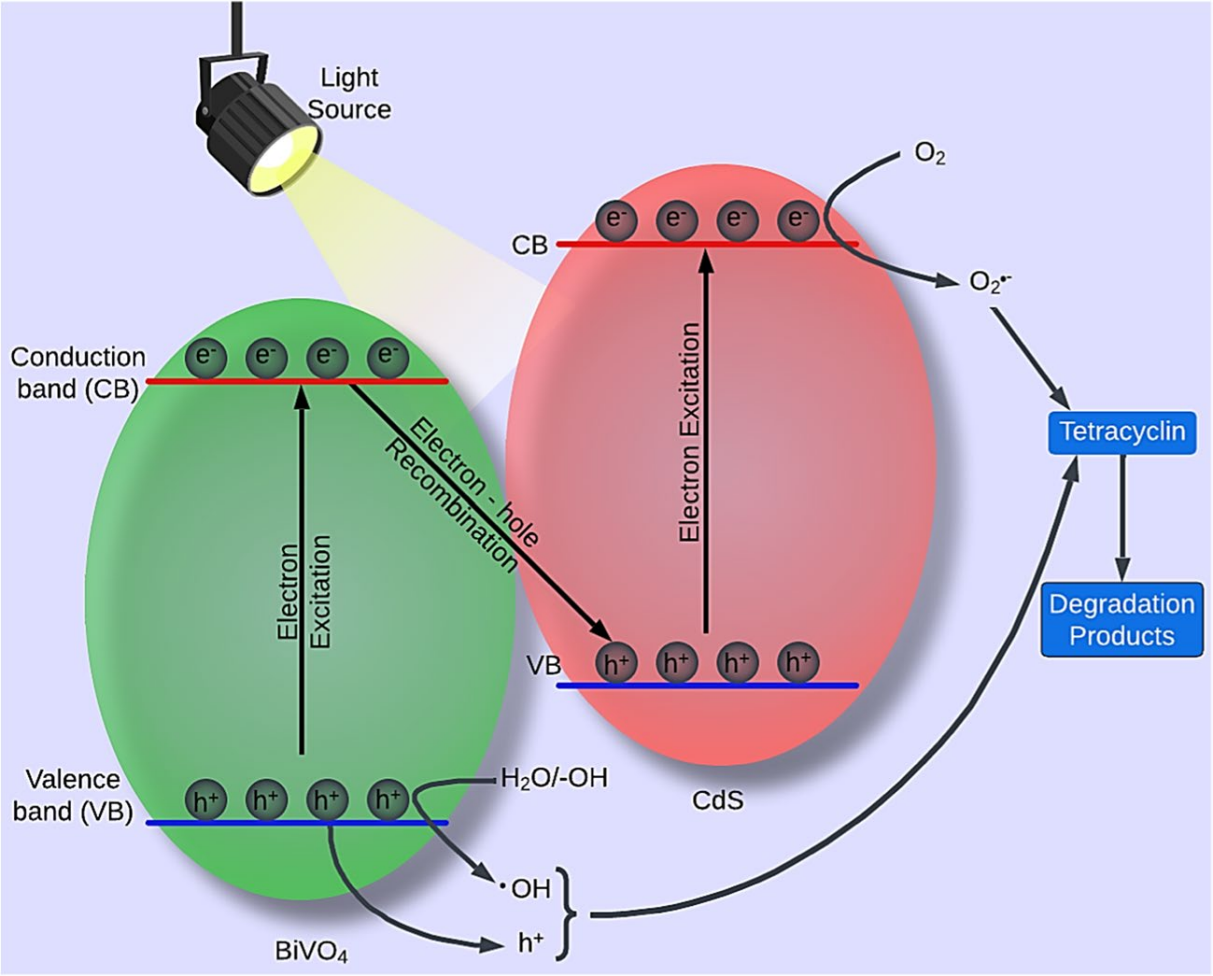
Z-scheme degradation pathway for dye removal

This test was also performed using sodium oxalate (Na_2_C_2_O_4_), BQ, and IPA to quench *h*^+^, O_2_^•−^, and ^•^OH, respectively (Xu et al. 2021). In their study, *h*^+^ and O_2_^•−^ were the major reactive species for tetracycline degradation using Fe_3_O_4_/BiVO_4_/CdS heterojunction composite photocatalyst (Xu et al. 2021) (Fig. 4). Based on the heterojunction assumption, the CB position (*E*_CB_) of BiVO_4_ (0.34 eV) was relatively weaker than the O_2_/O_2_^•−^ redox potential (− 0.33 eV). Moreover, the VB potential (*E*_VB_) of CdS (1.81 eV) was greater than the redox potential of OH^−^/^•^OH (+ 1.99 eV) and ^•^OH/H_2_O (+ 2.38 eV) (Xu et al. 2021). Hence, the Z-scheme path (Fig. 5) could be employed to describe the electron migration from the BiVO_4_’s CB (0.34 eV) to the CdS’s VB (1.81 eV), followed by recombination with the holes of CdS.

A higher electron–hole separation rate and redox potential support the accumulation of electrons at CdS’s CB, reacting with dissolved O_2_ to produce O_2_^•−^. In parallel, the holes located in the BiVO_4_’s VB have enough oxidation capacity to (i) react with tetracycline directly and (ii) combine with H_2_O or OH^−^ to generate ^•^OH.

Mineralization is one of the main objectives of photocatalysis, describing the conversion of organic carbons associated with the dye molecule to CO_2_ and H_2_O (Eq. 24):

24 $${}^{\bullet }{\text{OH}}+\mathrm{ dye}\to {\text{intermediates}}\to {{\text{CO}}}_{2} +{{\text{H}}}_{2}{\text{O}}$$

Mineralization is an essential dye removal pathway because it could mitigate the generation of undesirable toxic intermediate by-products (Zheng et al. 2020). The BiOBr/Fe_3_O_4_/RGO photocatalyst showed a strong mineralization performance towards rhodamine B dye removal, where about 91.45% of total organic carbon (TOC) was converted to CO_2_ and H_2_O in only 60 min (Zheng et al. 2020). The composite exhibited a promising adsorption–photocatalysis synergistic effect, depicting its applicability for treating industrial effluents without generating secondary pollution.

**Fig. 6:**
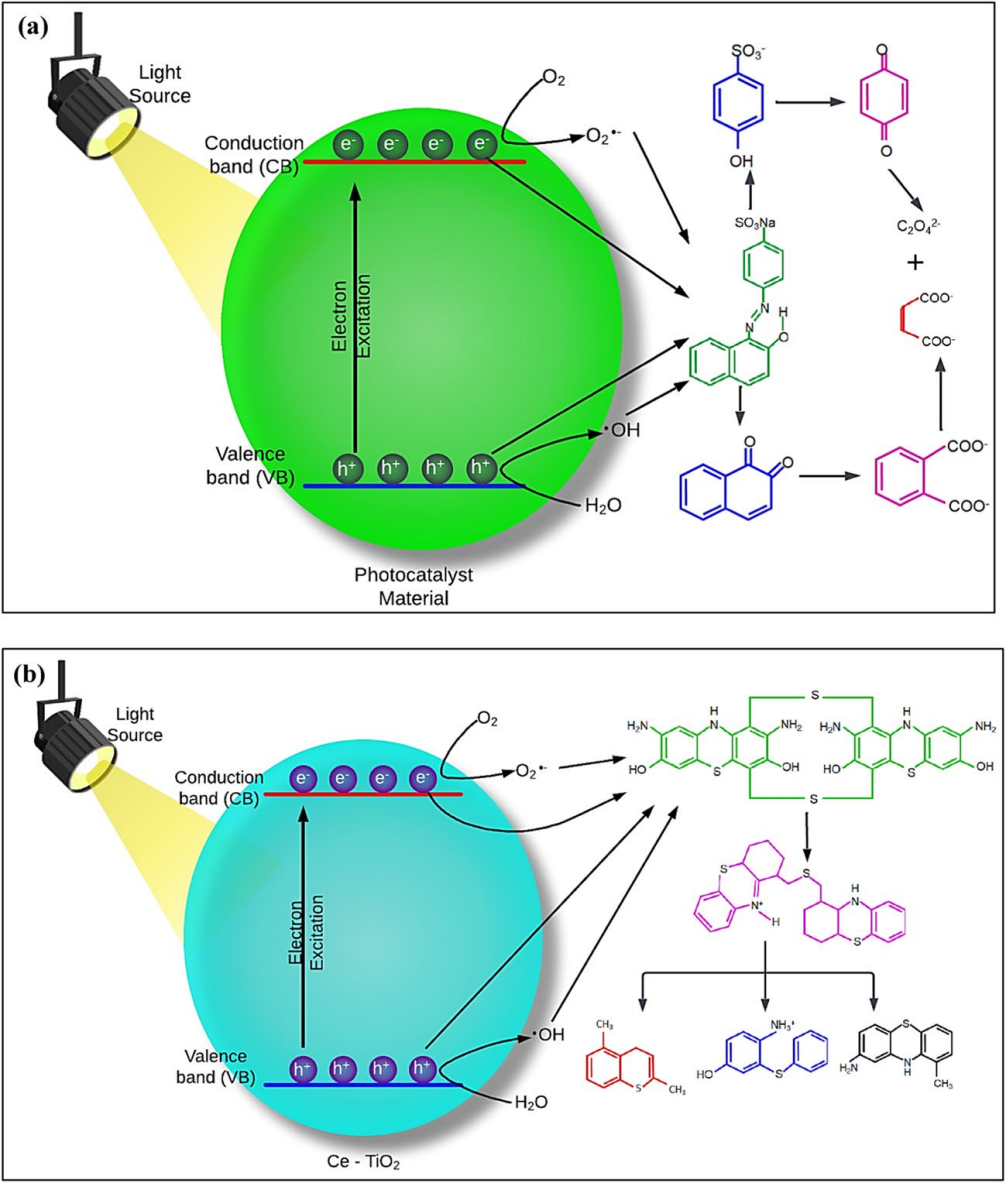
**a** General degradation by-products of azoic dyes; **b** degradation mechanism of sulfur black sulfur dye, adapted from Touati et al. (2019)

Real wastewater containing magenta dye was treated by photocatalysis using a TiO_2_-Fe_3_O_4_ composite material, where TOC was monitored before and after degradation (Pucar Milidrag et al. 2022). The decolorization and mineralization efficiencies were about 95% and 85%, respectively, revealing that dye decolorization could not achieve complete oxidation to CO_2_ and H_2_O. As such, various intermediates and by-products could be generated in the medium during dye decolorization. Monitoring and identification of such intermediates and by-products are essential not only to identify the removal pathway followed by dye degradation but also to recognize any toxic residuals. Liquid chromatography-tandem mass spectrometry (LC–MS) has been broadly used to exhibit superior and rapid separation efficiency, identifying the composition and structure of these intermediates. Moreover, the degradation pathway during the photocatalytic treatment of wastewater containing dyes could be semi-random, i.e., not following a sole specific route, depending on reaction conditions, and should be studied. Figure 6 shows the degradation mechanisms studied for an azoic dye and a sulphur dye adapted from literature (Touati et al. 2019).

## Management of magnetite-based photocatalysts after textile dye removal

### Reusability of magnetite-based photocatalysts

Magnetite-based photocatalysts have displayed several added advantages over other heterogeneous photocatalysts. For example, their magnetic nature allows them to be easily magnetically separated from a solution and reused (Upadhyay et al. 2021) for photocatalytic dye degradation (Table 4). Moreover, magnetic nanomaterials are relatively stable in nature, displaying minimum losses in photocatalytic performance over several cycles, and are able to work within the neutral pH range. For instance, a Fe_3_O_4_@SiO_2_@AgCl/Ag/Ag_2_S nanocomposite was synthesized and applied for photocatalytic degradation of methyl orange for 10 cycles, after which the degradation efficiency was reduced by only 14% (Mazhari et al. 2018). The textile wastewater treatment stability over 10 successive cycles was validated by revealing minor changes in the chemical composition, surface morphology, crystalline structure, and magnetic performance of the photocatalyst. Also, a ZnO/Fe_3_O_4_/g-C_3_N_4_ composite photocatalyst was prepared and used to treat wastewater containing methyl orange dye (Wu et al. 2019). After five cycles, the photocatalytic degradation percentage reduced by about 2.57%, and there were insignificant changes in the photocatalyst structure. These findings demonstrate that the number of cycles for photocatalyst reusability could vary according to several factors, such as the solute concentration, cycle period, photocatalyst stability, and material dosage.

**Table 4:** Reusability and regeneration of magnetite-based photocatalysts after textile dye removal

Photocatalyst	Dye	Number of cycles	Degradation efficiency, %		Ref
			After the first Cycle	After the last Cycle	
rGO-Fe_3_O_4_ /TiO_2_ nanocomposite	Malachite green (MG)	5	99	92	Bibi et al. (2021)
rGO-Fe_3_O_4_ /TiO_2_ nanocomposite	Methylene blue (MB)	5	97	90	Bibi et al. (2021)
Manganese dioxide-incorporated iron oxide three-dimensional nanoflowers (α-MnO_2_-Fe_3_O_4_)	Methylene blue (MB)	4	94.8	88	Rabani et al. (2022)
Manganese dioxide-incorporated iron oxide three-dimensional nanoflowers (α-MnO_2_-Fe_3_O_4_)	Crystal violet (CV)	4	93.7	89	Rabani et al. (2022)
Fe_3_O_4_/TiO_2_/Ag nanocomposites	Methylene blue (MB)	4	85	70	Fauzian et al. (2017)
TiO_2_@Fe_3_O_4_	Direct red 80 dye (DR-80)	3	100	90	Sadeghi et al. (2021)
ZnO/Fe_3_O_4_-sepiolite nanoplatform (ZnO/Fe_3_O_4_-SEP)	Methylene blue (MB)	3	100	-	Akkari et al. (2017)
Ternary magnetic ZnO/Fe_3_O_4_/g-C_3_N_4_ composite	Methyl orange (MO)	5	97.87	95.3	Wu et al. (2019)
Fe_3_O_4_@SiO_2_/MoO_3_/PDA-GO composites	Methylene blue (MB)	3	98	35	Vasallo-Antonio et al. (2021)
Ch.@Fe_3_O_4_@BiOCl microrobots (CFB)	Rhodamine blue (RhB)	4	95.6	90.4	Xu et al. (2022)
Fe_3_O_4_@TiO_2_@PDA/SiW11V-Ag multicomponent composite	Methyl orange (MO)	5	100	95	Wu et al. (2021)
MOF-1/GO/Fe_3_O_4_ nanocomposite	Methylene blue (MB)	4	95	92	Bai et al. (2020)
Magnetic BiOBr/Fe_3_O_4_/RGO composites	Rhodamine B (RhB)	4	96	90	Zheng et al. (2020)
Fe_3_O_4_@SiO_2_@AgCl/Ag/Ag_2_S nanocomposite	Methyl orange (MO)	10	99	85	Mazhari et al. (2018)
Fe_3_O_4_@MIL-100(Fe)	Methylene blue (MB)	5	99.77	≈99.77	Zhang et al. (2013)
g-C_3_N_4_/ZnO@Fe_3_O_4_ (Fe-heterojunction)	Rhodamine blue (RhB)	5	87	58.4	Yang et al. (2020)
Fe_3_O_4_@SiO_2_@TiO_2_ — 6%	Methyl orange (MO)	5	98	-	Mazhari and Hamadanian (2018)
Fe_3_O_4_@SiO_2_@TiO_2_	Methyl orange (MO)	5	93	85	Mazhari and Hamadanian (2018)
In_2_S_3_/ZnFe_2_O_4_ nanocomposites	Methylene blue (MB)	5	86.5	≈86.5	Zhao et al. (2020)

### Regeneration of magnetite-based photocatalysts

**Fig. 7:**
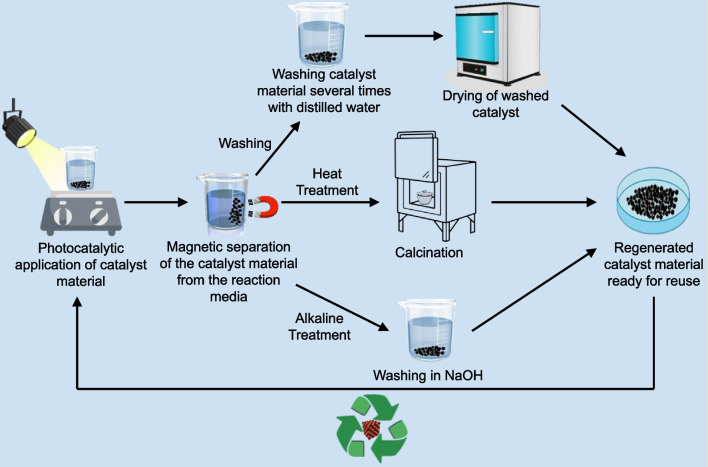
Magnetite-based photocatalyst regeneration procedures

The suitability and reusability of magnetite-based photocatalysts depend on performing a series of pre-treatment steps (Fig. 7) to detach the impurities and excess dye molecules.

The main objective of photocatalyst regeneration is to ensure that any possible blockage of the active sites by the degradation by-products is rectified. A study by Pucar Milidrag et al. (2022) represented the regeneration of a TiO_2_-Fe_3_O_4_ photocatalyst by separation from the reaction medium using an external magnetic field followed by extraction, washing several times with distilled water, and drying in an oven at 105 °C for 2 h. Once dried, the retrieved photocatalyst was then annealed in a muffle furnace at 200 °C for 2 h. Although the regeneration step is essential for removing the impeded impurities, further repeating this procedure might reduce the photocatalytic degradation efficiency due to material scattering and photo-corrosion. A similar study was carried out by Mazhari et al. (2018), regenerating a Fe_3_O_4_@SiO_2_@AgCl/Ag/Ag_2_S photocatalyst after photodegradation of methyl orange by separation from the dye solution using an external magnetic field. This step was followed by washing with ethanol and deionized water several times and then reusing for photodegradation of a methyl orange-rich solution.

Magnetite-based photocatalysts can also be regenerated by heat treatment. This involves subjecting the used photocatalyst material to high temperatures in order to remove any residual pollutant that may still be present on the catalyst material and causing performance losses (catalyst poisoning). For instance, in a study by (Hossein & Firoozeh 2019), a Fe3O4-PPY-NiO nanocatalyst material was regenerated after each cycle by first magnetically extracting the catalyst from the pollutant solution. This was followed by subjecting the extracted catalyst to heat treatment at 250 °C for 6 h before reapplying it for photocatalytic degradation. After five cycles, he catalyst material displayed a loss in activity of only 20%. Similarly, in a study by Wang et al. (2017), a γ-Fe2O3@SiO2@TiO2 photocatalyst material was subjected to photodegradation of rhodamine B dye for ten consecutive cycles without regeneration. This resulted in a reduction in removal efficiency from nearly 100% in the 1st cycle to about 35% in the tenth cycle. The catalyst material was then regenerated by heat treatment at 200 °C for 30 min. When reapplied for photocatalytic degradation after regeneration, the removal efficiency of the catalyst material was restored to about 100%.

Another catalyst regeneration technique that has been applied to magnetite-based photocatalysts is regeneration by alkaline treatment. In this technique, the catalyst material is washed with a solution of NaOH, dried, and reapplied for photocatalysis. In a study by Idris et al. (2011), the magnetically separable bead catalyst material was regenerated by subjecting the separated catalyst material to washing with a 0.01-M solution of NaOH for 1 h before being reapplied for Cr(VI) removal. The study alluded to the regenerative action of the NaOH to the formation of hydroxides of the pollutant by-products which can then easily detached from the catalyst material, resulting in the unblocking of the catalyst active sites. A γ-Fe2O3/SBA–15/TiO2 photocatalyst material was also regenerated similarly (Yu et al. 2015) after photocatalytic degradation of As(V). The catalyst material was washed in a 0.01-M solution of NaOH and reapplied for photocatalysis, where the catalyst material maintained a degradation efficiency above 90% after five cycles. In this case, the NaOH was able to remove As(V) embedded on the catalyst surface due to the overall negative charge that the catalyst obtains when washed with NaOH. Since arsenic is often present in the form of AsO43-, the negative charge in the catalyst and that on the AsO43- will result in repulsive forces; leading to the desorption of the As from the catalyst surface.

## Reuse of treated dye-laden wastewater in the textile industry

Textile manufacturing processes consume large quantities of water, reaching approximately 79 billion cubic meters per annum in 2015 (Pinto et al. 2022). The reuse of treated textile effluents in companies and manufacturing industries for dyeing fabrics is an essential step in maintaining water resource conservation. However, because the effluents of this industry contain dyes, solvents, organic acids, defoamers, reagents/buffers, and washing chemicals, appropriate wastewater treatment methods are required. The quality standards of water used in the textile industry (e.g., for dyeing, cleaning, printing, and finishing) should be better than the water disposal threshold regulations. Wastewater reuse scenarios employed to fulfil the water consumption requirements in the textile finishing industries in Europe were declared (Pinto et al. 2022; Vajnhandl & Valh 2014). Their studies proposed that the integration of multiple treatment technologies (microfiltration and nanofiltration membrane system, coagulation/flocculation/sedimentation, and advanced oxidation processes) was suitable to reduce more than 40% of freshwater consumption by the textile industrial sector. Table S1 lists the applications of treated effluents in the textile industries. Magnetite-based photodegradation processes have displayed sufficient dye and colour removal abilities of up to 100% (Akkari et al. 2017; Sadeghi et al. 2021; Wu et al. 2021) when applied to synthetic and real textile dye-laden waters. Because of their potential to mineralize organic pollutants in waters (Gnanaprakasam et al. 2015; Vinu et al. 2010), magnetite-based photocatalysts could also be used to significantly reduce COD, organic compounds, toxic metals content, and plasticizer content (Khan et al. 2019) of textile wastewaters. The final effluent characteristics are then compared with the allowable ranges before its reuse. Magnetite-based photodegradation is an ideal low-cost, non-toxic, and efficient treatment technique that can be combined with simple treatment processes, such as water softening, pH adjustments, and sedimentation, to treat dye-laden wastewater. The photodegradation effluent can also be reused in various textile processes.

## Economic performance of dye removal by magnetite-based photodegradation

The application of magnetite-based photocatalysts for treating dyes-laden wastewater was economically assessed. This step was performed to explore the feasibility of the medium-scale (influent of 10 m^3^/day) application of the dye-laden wastewater treatment process. The analysis included estimations of capital costs, operating costs, and revenues/earnings.

### Capital costs

The initial investments considered in this analysis represented the capital costs of several items. These items shared land footprint, machinery purchases, electrical wiring and piping works, construction expenses, and other amortized costs.

The required volume of the treatment tank (*V*_*c*_), as calculated by Eq. 25 (Alalm & Nasr 2018), was used to estimate the construction costs:

25 $${V}_{c}= \frac{Q}{D} \times \frac{{t}_{t}}{{t}_{w}}$$

where *Q* is the average volume of wastewater treated annually (m^3^/year), *D* is the number of working days per year (assuming 300 day/year), *t*_*t*_ is the reaction time (e.g., 10 min), and *t*_*w*_ is the number of plant operation hours per day (e.g., 720 min/day).

The estimated *V*_*c*_ value was further used to calculate the construction costs (CC) by Eq. 26 (Ansari et al. 2019):

26 $$CC= {V}_{c}\times {C}_{P}$$

where *C*_*p*_ is the cost price per m^3^ of a complete photoreactor unit (retrieved from suppliers’ quote).

The capital costs of other items were estimated using CC in the formulae derived from previous studies (Mahamuni & Adewuyi 2010), as given in Table 5. The land purchasing cost was estimated at 1.5% of all other capital investment costs.

**Table 5:** Cost–benefit analysis for photocatalytic degradation of dyes using magnetite – based photocatalysts (flowrate = 10 m3/day)

	Description	Justification	%	Unit	Cost
Capital costs	Land acquisition	≈ 1.5% of all other capital costs	1.48	$	4882.01
	Photocatalysis reactor tank	CC/0.4	11.30	$	37,321.18
	Pumps for treated effluent and used-up catalyst removal		4.84	$	15,994.79
	Treated water tank construction		3.23	$	10,663.19
	Used-up photocatalyst tanks		1.61	$	5331.60
	Pipes and valves		0.97	$	3198.96
	UV lamps		6.46	$	21,326.39
	Stirrer motor		3.87	$	12,795.83
	Catalyst synthesis chambers		22.59	$	74,642.36
	Washing and filtration tank		2.23	$	7377.05
	Furnace		1.98	$	6533.33
	Construction works, including installations of tanks, piping, valves, and electrical works and site works	CC	12.91	$	42,652.78
	Contractor charges	0.15 (CC + CC/0.4)	6.78	$	22,392.71
	Engineering consultancy charges	0.15 (0.15 (CC + CC/0.4))	7.80	$	25,751.61
	Contingencies and other	0.2 (0.15 (0.15 (CC + CC/0.4)))	11.95	$	39,485.81
	Total capital costs		100.00	$	330,349.61
Operating costs	Chemicals	420.21 kg/year × $16.67/kg	22.43	$/yr	7004.90
	Electricity consumption by UV lamps, pumps, stirrer motor ($739.8451/1000 gallons)	$0.16/kWh × 16.68 kWh/m^3^ × 3000 m^3^/year	25.64	$/yr	8004.92
	Treatment and catalyst regeneration water	$0.45/m^3^ × 10 m^3^/day × 300 days	4.32	$/yr	1350.00
	Repair and maintenance of all equipment	≈ 1.5% of all other capital costs	15.87	$/yr	4955.24
	Government taxes	≈ 1% of all other capital costs	10.58	$/yr	3303.50
	Workers’ remuneration	≈ 2% of all other capital costs	21.16	$/yr	6606.99
	Total operating costs	-	100.00	$/yr	31,225.55
	Unit operating cost	Total operating cost ÷ (10 m^3^/day × 300 days)	-	$/m^3^	10.41
Revenues	COD removal	-		$/yr	2698.08
	Treated water reuse on-site	-		$/yr	463.68
	Dye colour removal	-		$/yr	78,800
	Total revenues			$/yr	81,961.76
	Net profits	Total revenues $/year − operating costs $/year		$/yr	50,736.21
	Payback period	Total capital costs ($) ÷ net profits ($/year)		Years	6.51

### Operating costs

The operating costs are used to define the expenses incurred to continuously run the treatment of dyes-laden wastewaters using magnetite-based photocatalysts. These costs are associated with chemicals, electricity, water, repair and maintenance, taxes (statutory obligations), and other periodic expenses. The cost of chemicals was calculated by Eq. 27 (Hamdy et al. 2018):

27 $${{\text{Cost}}}_{{\text{chemicals}}}= {C}_{i} \times {P}_{i}$$

where *C*_*i*_ is the quantity of chemicals and reagents required per annum (kg/year) and *P*_*i*_ is the cost of the chemical per unit weight ($/kg).

In this calculation, it was assumed that the optimum photocatalyst concentration was 700 mg/L, and it was regenerated and recycled up to 5 times before being carefully discarded and replaced with a fresh catalyst.

The electricity consumption costs were calculated using a consumption rate of 0.16$/kWh (Dadebo et al. 2022). The electricity consumption for treating 1 m^3^ (electrical energy per order, *E*_EO_) of wastewater was computed using the formula given by Eq. 28 (Kumari et al. 2023; Olya et al. 2016). The consumption per m^3^ and the consumption costs were then used to calculate the annual cost associated with electricity consumption.

28 $${E}_{EO}=\frac{38.4\times P }{V\times k}$$

where *P* is the power of all electrical equipment (estimated as 100 W), *V* is the volume of treated water per day (L), and *k* is the reaction rate constant (estimated from plot of ln (*C*/*C*_*o*_) *vs*. time).

The costs of water utilized to synthesize and regenerate the photocatalyst, prepare an adapted pH medium, and clean the tools and devices are also included in the operating costs. For instance, 300 mL of water, with a water tariff of 0.45$/m^3^ (Plappally & Lienhard 2012), could be used to prepare 1 g of catalyst. The repair and maintenance costs were estimated at 1.5% of the capital costs (Ansari et al. 2019). The taxes and workers’ remuneration costs were estimated at 1% and 2% of the total capital costs, respectively.

The total operational costs were expressed by electricity consumption (25.64%), chemical utilization (22.43%), water use (4.32%), and others, giving a total running cost of $10.41/m^3^. A cost analysis (UV light tubes, air sparger, and nano-material preparation) for applying TiO_2_ in the photocatalytic degradation of Remazol Red dye showed an operating cost of $0.29/L ($290/m^3^) of treated textile industry effluent (Pipil et al. 2022). In another study (Chawla et al. 2020), the costs of eliminating basic yellow 28 dye using p25-TiO_2_ and ana-TiO_2_ were estimated and compared. The operational costs were estimated from the prices of energy and materials, showing Rs. 5090/m^3^ ($61.09/m^3^) and Rs. 2900/m^3^ ($34.80/m^3^), respectively (Chawla et al. 2020). Another study utilized TiO_2_ for the photocatalytic removal of pesticides from wastewater (Gar Alalm et al. 2015), revealing an operational COD removal cost of €7.98/m^3^ ($8.73/m^3^).

### Revenues

The weight of COD removed could be used as an essential criterion to estimate the profits of using the photocatalytic process to reduce environmental pollution. This profit was equivalent to $0.14 for removing a kg COD from industrial effluents, giving 2698.08 $/year. This amount is added to the revenue of the project cash flow, representing economic benefits derived from the initiation of pollution prevention strategies (Ansari et al. 2019). In addition to COD, colour removal was estimated as the removal of nitrogen and phosphorous. Removing 1 kg of each of nitrogen and phosphorous from the textile effluents could add about 8 and 30 $ to the total profits (Ansari et al. 2021). The benefit of onsite reuse of the treated effluent was also added to the project revenue. This value is equivalent to $0.45 per m^3^ of reused treated water (Dadebo et al. 2022).

### Profitability criterion

The economic feasibility of the dye photodegradation process was expressed by the project payback period. The project net profit was estimated from revenue (81,962 $/year) minus operating cost (31,226 $/year), giving 50,736 $/year. The payback time reached about 6.5 year, as calculated from capital cost (330,350 $) ÷ net profit (50,736 $/year). By assessing the cash flow, the revenue gains from pollutants’ removal would recover the prices invested in purchasing the photodegradation project facilities. Moreover, a shorter project payback period (below the project lifetime of 15 year) would be expected due to reusing the exhausted photocatalyst for about five regeneration/reuse cycles.

## Achievable sustainable development goals (SDGs) from the photocatalyst technology implementation

The 17 sustainable development goals (SDGs) are a set of goals that include respective targets adopted by the United Nations (UN) member states, achieving world peace and prosperity now and in the future. The goals are integrated to end poverty, improve health and education, reduce inequalities, trigger economic growth, tackle climate change, and preserve oceans and forests. The UN SDGs (Table 6) can be grouped into the social, economic, and environmental pillars of sustainability. This section discusses the SDGs that can be achieved by synthesizing magnetite-based photocatalysis for further applications, including dye-laden wastewater treatment.

**Table 6:**
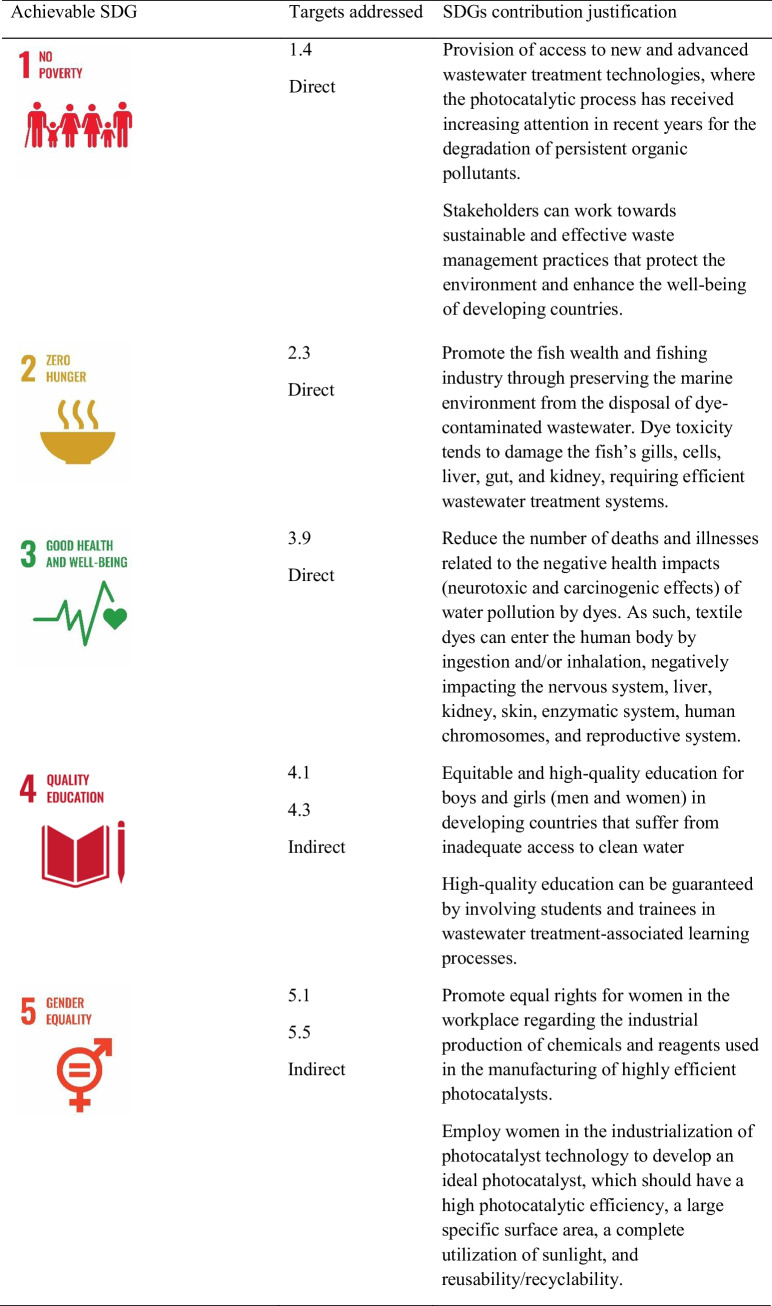

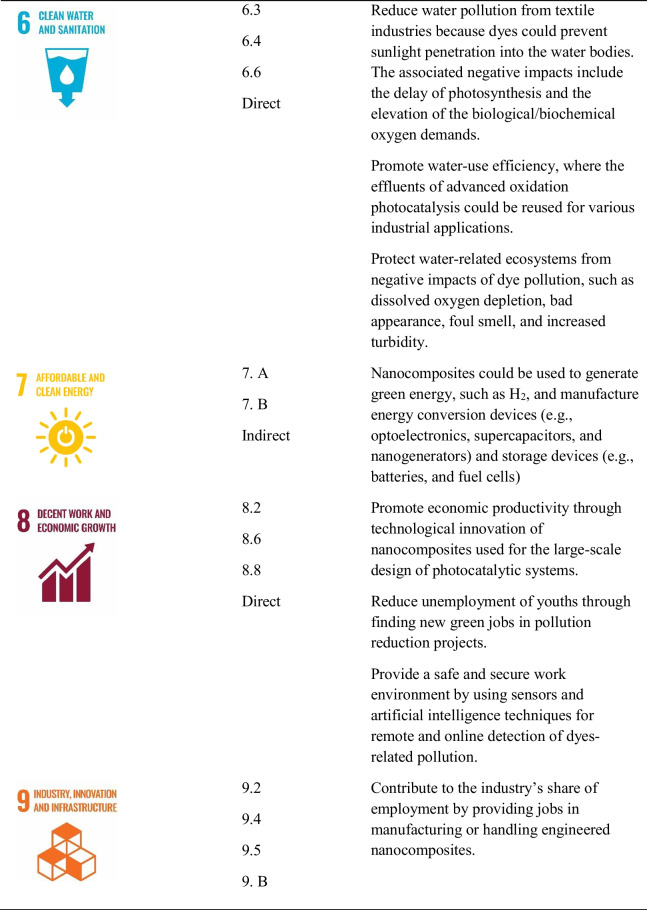

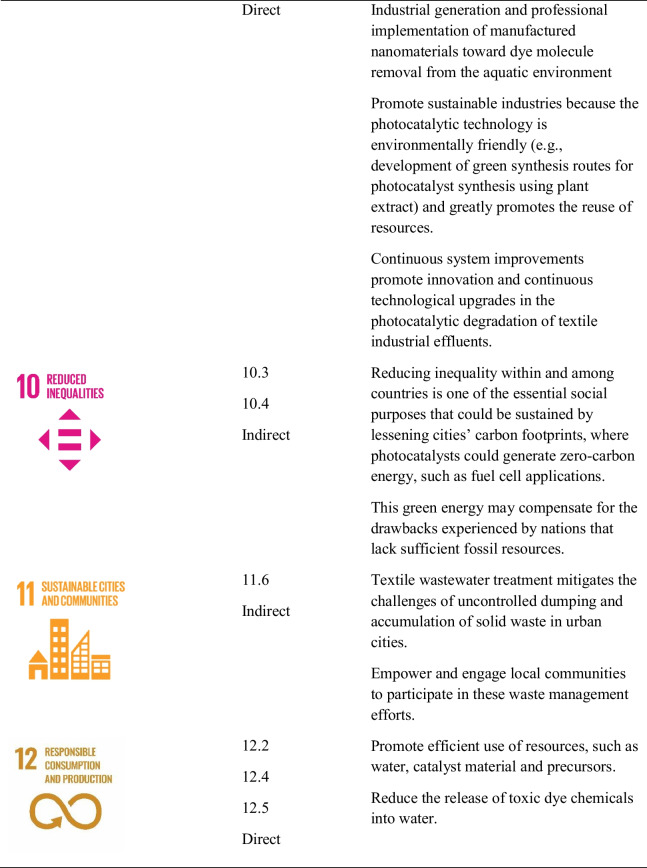

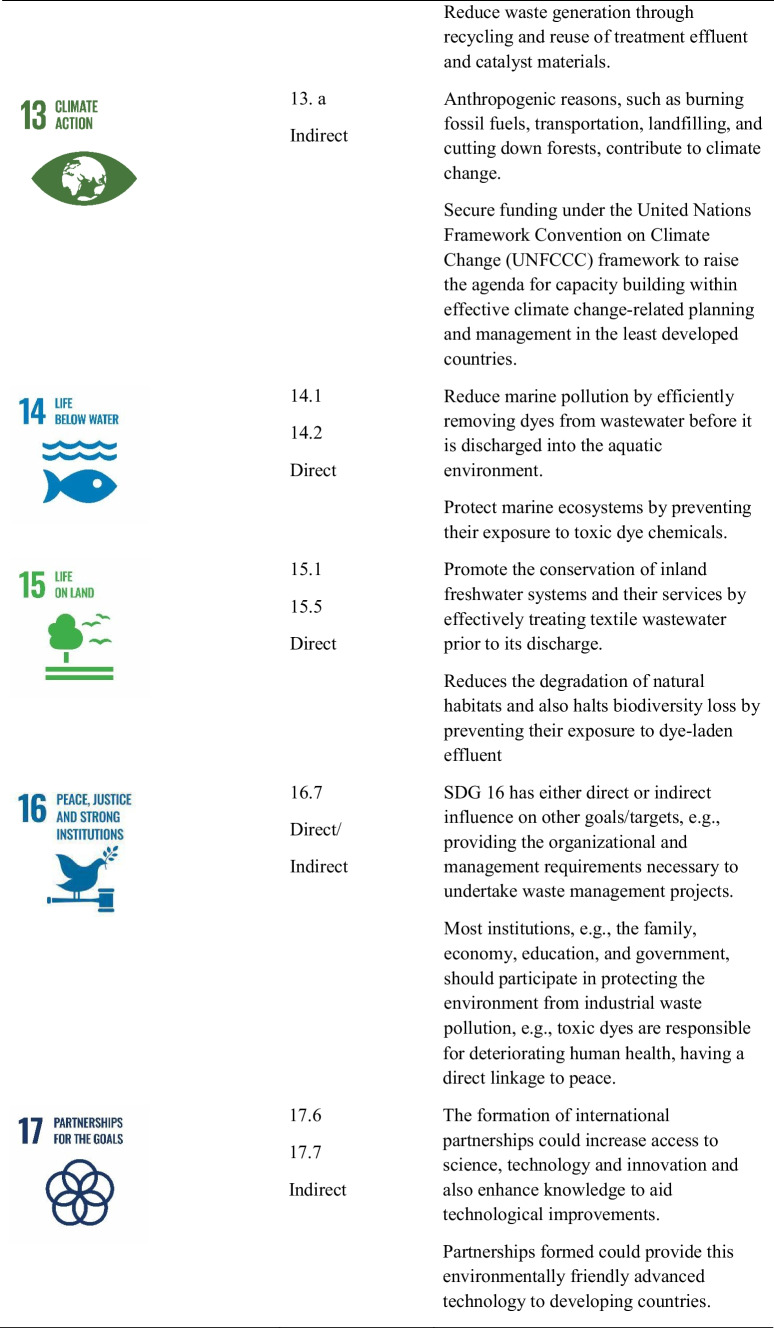
Achievable SDGs, their associated targets and the contributions made by magnetite-based photocatalysts and their application in treating textile industry effluents

### Social SDGs

Recent crises, such as the COVID-19 pandemic, rising inflation, and several wars, have elevated the poverty rate and negatively impacted some natural resources such as water. Water is a vital resource required to drive development, and it is crucial for operating various manufacturing industries. For instance, water is utilized at several stages (e.g., bleaching, dyeing, washing, and cooling of equipment) in the textile industry. Magnetite-based photocatalysis could mineralize complex compounds, such as dyes in aqueous solutions, into simpler molecules with less toxicity. This property enables the reclamation of dye-laden wastewater into water of acceptable quality, which can be reused in various human activities, supporting social infrastructure development. Reusing the treated effluents in the industrial sector makes freshwater more available and affordable to society (SDG1: No Poverty). This benefit supports developing countries that do not have access to clean water and sanitation. Recently, several actions have been directed towards resolving food‐web structure and ecosystem function, maintaining SDG2 “Zero Hunger.” For example, reusing the treated effluent in irrigation would boost farming activities and support the agricultural sector.

Moreover, treating the industrial effluents protects the fish species richness from dye toxicity, imposing positive impacts on marine fisheries production and fish farming industries. As such, dye-laden wastewater is toxic to marine life because it slows down photosynthetic processes, reducing the DO levels in the water. This pattern also avoids humans’ exposure to harmful dye chemicals, thereby preventing illness or even death (SDG3: Good health and wellbeing). Scholars and trainees in developing countries can be involved in environmental-related training programs to learn about the applications of wastewater treatment techniques such as magnetite-based photocatalysis (SDG4: Quality Education). Moreover, improved water quality can reduce the risk of waterborne diseases, increasing the number of students (boys and girls) attending classes in the school. The industrial application of magnetite-based photocatalysis for removing dyes can provide job opportunities for women in both low-level and managerial positions (SDG5: Gender Equality). Implementing magnetite-based photocatalysis in low-income nations requires technical assistance and financial aid from more developed countries in accordance with World Trade Organization agreements. This action encourages equality among countries regarding the rights to strengthen and sustain cleaner and greener technologies (SDG10: Reduced inequalities). Transferring the knowledge and skills about advanced wastewater treatment technologies to countries that have complex water security policy challenges is an essential objective for the implication of SDG16 “Peace, justice and strong institutions.” This goal also promotes peaceful relations between the developed and developing nations through improving sustainable planning of the water-food nexus. The formation of international partnerships supports the countries identified with serious water resources shortage and environmental pollution concerns. Moreover, it is essential to maintain strong partnerships between developed and developing nations to alleviate the financial burden related to the initial investment of the magnetite-based nanocomposite manufacturing processes (SDG17: Partnerships for the goals).

### Economic SDGs

The manufacturing of magnetite-based photocatalysts and nanocomposites influences several economic-related SDGs. For instance, SDG7, “Affordable and clean energy,” can be endorsed by applying the synthesized nanomaterials in the energy storage and conversion facilities (Muzhanje & Hassan 2023). For instance, phase change materials were enhanced by Al_2_O_3_ and CuO nanoparticle supplementation and then studied for their applications towards energy sustainability (harvest-free and/or waste thermal energy). Nanomaterials are also employed in the energy sector organizations by enhancing the solidification, heat transfer, and melting behaviours of the phase change materials (Muzhanje et al. 2023). As such, this approach can address SDG7 by providing efficient, affordable, and sustainable solutions to face the problems associated with conventional heating and cooling systems.

Moreover, the photocatalytic H_2_ production approach provides a clean and renewable energy source, such as H_2_ energy application as a future alternative transport fuel. Therefore, photocatalytic nanomaterials can be employed to ensure access to reliable, sustainable, and modern energy, in addition to their existing technologies in wastewater treatment. The applications of photocatalytic technology in environmental safety open more avenues for offering new green jobs (i.e., maintaining SDG8: Decent work and economic growth by employment in environmental-friendly projects). The innovation and industrialization of photocatalysis can open new opportunities for many employees who lost their jobs and companies forced to close down their businesses during the pandemic. Reducing water pollution plays an important role in addressing economic growth and development (SDG9: Industries, innovation and infrastructure). For example, poor water quality reduces labour productivity (health issues), deteriorates the quality and quantity of food production, and negatively impacts the aquaculture and fisheries sectors. Photocatalysis technology can counter the shortfalls associated with conventional methods implementation in wastewater treatment regarding sludge transfer and open dumping in various environmental matrices.

Moreover, wastewater treatment is one of the waste management strategies used to reduce per capita environmental pollution in cities. Addressing these concerns makes cities and human settlements inclusive, safe, resilient, and sustainable (SDG11: Sustainable cities and communities). The strong magnetic property of magnetite-based photocatalysts facilitates their easy separation from the reaction medium, followed by their regeneration and re-application. This property reduces the introduction of new catalysts into the system, promoting sustainable consumption of raw materials. In parallel, this technology lessens the amount of waste generated (i.e., due to material reuse several times), ensuring sustainable consumption and production patterns “SDG12.” Furthermore, the treated effluents could be reused in the textile industry (e.g., cooling of mechanical parts and cleaning and washing of the facilities; see Table S1), reducing the overall water consumption patterns.

### Environmental SDGs

Applying magnetite-based photocatalysis for textile dye removal shows great progress in sustaining clean water provision, permitting more people to access clean water and water facilities (SDG6: Clean water and Sanitation). This wastewater treatment approach also attempts to conserve and sustainably protect marine resources and ecology from industrial pollution (SDG14: Life below water) because the dye molecules are absorbed by the fish’s gills and skin, followed by bioaccumulation and biotransformation. Because this process generates fewer quantities of sludge compared with conventional biological systems, it does not cause considerable solid waste pollution (SDG15: Life on Land). As such, the landfilling of textile dyeing sludge poses a threat to the terrestrial environment and ecosystems due to the presence of a wide range of pollutants and hazardous chemicals. The improper consideration of synthetic dyes (e.g., fibres, plastic, polyester, and yarns) increases the possibility of natural habitat degradation and biodiversity loss. Mitigating air pollution by photocatalysis has also been reported, where the CO_2_ greenhouse gas could be reduced with H_2_O vapor to generate hydrocarbons and syngas. Converting the greenhouse gas into beneficial sustainable energy fuel (e.g., CH_4_ and H_2_) and utilizing the generated fuel to alleviate dependency on fossil fuels are the main routes used to meet the targets of SDG13, “Climate action.”

## Barriers facing the application of magnetite-based photocatalysis

**Fig. 8:**
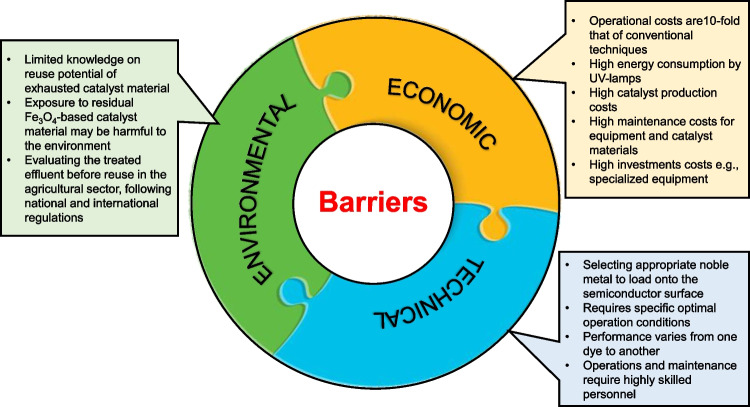
Barriers facing the sustainability of magnetite-based photocatalysis applications in treating textile industry effluents

Applying magnetite-based photocatalysis to treat dye-laden wastewater has great potential in protecting the environmental dimensions. However, certain challenges should be addressed to fulfil the other pillars of sustainability (economic and social). According to the SDGs concept, these challenges can be grouped as follows (Fig. 8):

### Technical barriers

Technical challenges refer to the factors involved in the functionality/complexity, up-scale feasibility, infrastructure, equipment, and other tools used to operate the photocatalysis system. For instance, although magnetite is considered a semiconductor material that possesses good photocatalytic, adsorption, and magnetic properties, its operation suffers from oxidation during generation and cleaning. Combining the magnetite material with other semiconductors or noble metals is one of the technical solutions used to avoid material oxidation, inadequate light utilization, and *e*^−^/*h*^+^ rapid recombination. However, the semiconductor materials should be rigorously tested and adequately selected because the magnetite phase has the potential to act as a charge carrier recombination centre, reducing the photocatalytic activity of the integrated material. Moreover, the magnetite-based photocatalysis process should be operated under specific pH and time ranges to maintain the best dye degradation schemes within shorter periods, especially for up-scale applications suffering from high fluctuations in wastewater characteristics.

Moreover, employing the same and generalized catalyst to eliminate wide concentrations of different dyes entails technically inaccurate photodegradation performances. For example, a ternary magnetic ZnO/Fe_3_O_4_/g-C_3_N_4_ composite could degrade about 98% of methyl orange and Alizarin yellow R, but this efficiency was reduced to 83.35% for orange G elimination (Wu et al. 2019). In particular, magnetite-based photocatalysis requires highly skilled and competent personnel for periodic repair, monitoring, and maintenance requirements, ensuring the functionality of all operational parameters at their optimum levels.

### Economic barriers

Referring to the cash flow given in Table 5, the operation and maintenance cost for treating 1 m^3^ of dye-laden wastewater using magnetite-based photocatalysis reached up to $10.41. Although this price is lower than that ($17.745/m^3^) used to treat dibutyl phthalate-laden wastewater by Fe_3_O_4_@PAC as a magnetic nano-composite (Nozari et al. 2022), photocatalysis is still considered an expensive process. As such, magnetite-based photocatalysis is almost 10 times more expensive compared with conventional wastewater treatment techniques, such as anaerobic/aerobic reactors and stabilization ponds ($1.10–$1.46/m^3^) (Sekandari 2019). Additionally, although solar radiation is abundant in nature and freely available, artificial sources of light (UV radiation) might also be required to possess the photon energy (according to the photocatalyst’s narrow band gap) for efficient dye degradation. Moreover, synthesizing large quantities of magnetite-based photocatalysts for field (up-scale) wastewater treatment applications can be economically infeasible because some of them still need rare or expensive noble metals. The photocatalyst’s manufacturing processes entail complex reactions and require specialized equipment and tools, ultimately raising investment costs (Kuspanov et al. 2023). Despite its recyclability for up to five consecutive runs, this photocatalyst is prone to oxidation and corrosion during handling and transfer. The associated photocatalyst deterioration requires frequent monitoring and maintenance to ensure material effectiveness. Furthermore, some expensive materials such as silica and titanium are used to coat and modify the magnetite-based photocatalysts, increasing their stability for treating industrial effluents under pH-varying conditions.

### Environmental barriers

The disposal of exhausted magnetite-based photocatalysts is a major environmental challenge that should be addressed to avoid secondary pollution. As such, this photocatalyst might adsorb some heavy metals and persistent pollutants that could transfer to the environmental domain and then to the human food chain. Thermal treatment (e.g., incineration, pyrolysis, and gasification) is one of the reliable strategies used to manage the utilized photocatalyst. Moreover, the treated effluent should be technically evaluated and characterized for its physicochemical and bacteriological properties because the reuse of treated water should comply with national and international regulations. For instance, the mutagenic and carcinogenic potential of textiles is harmful to human health, and the dye molecules and photocatalyst fine powder could affect plant roots during cultivation with treated textile wastewater, damaging soil quality and plant growth. A study was conducted to understand the exposure effect of magnetite-based nanocomposites on zebrafish embryos and larvae, depicting morphological and physiological alterations at a concentration of 1000 µg/mL (Guillén et al. 2022). However, the genotoxicity of magnetite-based photocatalysts varies depending on the type of material used to prepare the heterogenic composite. Therefore, the risk of toxicity resulting from the accumulation of residual photocatalyst material not separated from the treated effluent should be fully understood before the up-scale application of magnetite-based photocatalysis.

## Conclusion and future perspectives

This review succeeded in providing insights into the techno-economic feasibility and sustainability of synthesizing magnetite-based photocatalysts and their applications in textile industry effluent treatment. The dye degradation pathways were explored via electron excitation and transfer, water ionization, oxygen ionosorption, and superoxide protonation. Fe_3_O_4_-contained photocatalysts were distinguished by their shape (e.g., spheres, rods, and flowers) and composition (composites, hybrids, or single-phase particles). These types were defined based on the synthesis methods, e.g., hydrothermal, sol–gel, and ultrasonication. The dyes’ removal mechanisms depicted a high correlation with material characterization, regarding the crystalline structure, surface molecular composition, material morphology, and magnetic moment centres. The photocatalytic performance was highly influenced by substrate pH, reaction time, dye concentration, catalyst dosage, and interfering ions and humic acid. The magnetite-based photocatalysts could be regenerated and reused over eight cycles, and the quality of treated effluents could comply with the regulation standards for wastewater reuse in the textile manufacturing industries. For instance, the best magnetite-based photocatalyst was found to be Fe3O4@SiO2@AgCl/Ag/Ag2S nanocomposite as it was recycled up to 10 times under optimal conditions (catalyst dosage = 1200 mg/L, neutral pH, methyl orange concentration = 10 mg/L and time = 60 min).

It should be noted that the composite displayed minimal losses in performance of only 10% ± 4 even after ten consecutive cycles. The study also demonstrated the techno-economic feasibility of scaling up magnetite-based photocatalysis for textile wastewater treatment, giving a payback period shorter than the project’s lifetime. The benefits, as mentioned above, displayed strong interlinkages with the social, economic, and environmental pillars of sustainability, identifying and mitigating the impact of barriers to meet the SDGs strategy.

To increase the feasibility and profitability of the large-scale application of magnetite-based photocatalysis, more research should focus on (i) the possible recycling of the photocatalyst material in the industrial sector for manufacturing magnetic products; (ii) the synthesis of new photocatalysts that could treat dye-rich wastewater under wider pH range, reducing the costs of chemicals required for pH adjustments; (iii) studying the effect of reaction temperature on the performance of magnetite based photocatalysts; (iv) designing cost-effective photoreactors that could utilize natural sunlight for operations, reducing electricity utilization by UV lamps; and (v) getting supports from the international funding agencies to comply with environmental sustainability (e.g., green finance).

## Supplementary Information

Below is the link to the electronic supplementary material.

Supplementary file1 (DOCX 2060 KB)

## Data Availability

The data that support the findings of this study are available within the article and/or its supplementary materials.

## Abbreviations

MB: Methylene Blue
Fe_3_O_4_: Magnetite
AOP: Advanced oxidation process
XRD: X-ray diffraction analysis
FTIR: Fourier Transform infrared spectroscopy
SEM: Scanning electron microscopy
VSM: Vibrating sample magnetometer
TOC: Total organic carbon
COD: Chemical oxygen demand
ROS: Reactive oxygen species
PZC: Point of zero charge
VB: Valence band
CB: Conduction band
rGO: Fe_3_O_4_/TiO_2_ nanocomposite–reduced graphene oxide-based iron oxide modified titania
3D α-MnO_2_-Fe_3_O_4_: Manganese dioxide-incorporated iron oxide three-dimensional nanoflowers
Fe_3_O_4_/TiO_2_/Ag: Iron oxide and titanium dioxide nanocomposite with additional silver metal
TiO_2_@Fe_3_O_4_: Magnetic titanium dioxide nanoparticles
ZnO/Fe_3_O_4_-SEP: Zinc oxide/magnetite-sepiolite nanoplatform
ZnO/Fe_3_O_4_/g-C_3_N_4_: Ternary magnetic zinc oxide/magnetite/graphite-like carbon nitride composite
g-C_3_N_4_/ZnO@Fe_3_O_4_: Graphitic carbon nitride/zinc oxide on magnetite
Fe_3_O_4_@SiO_2_@TiO_2_: Titanium dioxide on silicon dioxide on magnetite
In_2_S_3_/ZnFe_2_O_4_: Indium sulphide/zinc ferrite nanocomposites
Fe–p-C_3_N_4_: Iron-loaded polymeric carbon nitride
Fe_3_O_4_/SnO_2_: Magnetite/tin oxide nanocomposites
Zn_0__.__5_Fe_3-0.5_O_4_: Zinc-doped spinel magnetite nanoparticles
TiO_2_/Fe_3_O_4_: Titanium dioxide/magnetite nanoparticles
Fe_3_O_4_/BiVO_4_/CdS: Magnetite/bismuth vanadate/cadmium sulphide heterojunction composite
Fe_3_O_4_/SiO_2_/TiO_2_: Pomegranate-like magnetite/silicon dioxide/titanium dioxide composite microspheres
Fe_3_O_4_/ZnO/Si_3_N_4_: Magnetite/zinc oxide/silicon nitride nanocomposite
g-C_3_N_4_/ZnO@Fe_3_O_4_: Graphitic carbon nitride/zinc oxide on magnetite
Ch.@Fe_3_O_4_@BiOCl microrobots: Ternary biohybrid photocatalytic microrobot based on Chlorella (Ch.) cells
Fe_3_O_4_@TiO_2_@PDA/SiW_11_V-Ag: Silver nanoparticles on polydopamine and mono-vanadium substituted silicotungstate co-encapsulated titanium dioxide on magnetite microspheres
MOF-1/GO/Fe_3_O_4_: Neodymium metal–organic framework/graphene oxide/magnetite nanocomposite
Magnetic BiOBr/Fe_3_O_4_/RGO: Bismuth oxyhalide/magnetite/reduced graphene oxide composite
Fe_3_O_4_@SiO_2_@AgCl/Ag/Ag_2_S: Silver chloride/Silver/silver sulphide on silicon dioxide on magnetite core–shell nanocomposite
Fe_3_O_4_@MIL-100(Fe): Metal–organic framework on magnetite core–shell microsphere
Fe_3_O_4_@SiO_2_/MoO_3_/PDA-GO: Silicon dioxide on magnetite/molybdenum trioxide/graphene oxide polydopamine matrix
