# Supplementary material for: Advancing sustainable water treatment strategies: harnessing magnetite-based photocatalysts and techno-economic analysis for enhanced wastewater management in the context of SDGs
Source: Environ Sci Pollut Res Int. 2024 Mar 12;32(49):28159–95. doi: 10.1007/s11356-024-32680-9 (PMC12711978; doi:10.1007/s11356-024-32680-9)
Supplement: Supplementary file 1 — Supplementary file1 (DOCX 2060 KB) [file 11356_2024_32680_MOESM1_ESM.docx]

*Supplementary material for*

**Advancing Sustainable Water Treatment Strategies: Harnessing Magnetite-Based Photocatalysts and Techno-Economic Analysis for Enhanced Wastewater Management in the Context of SDGs**

# **Bibliometric and review methods**

The terms “magnetite AND photodegradation AND dyes” were used as the main keyword search strings in the SCOPUS online database (<https://www.scopus.com/search>, accessed on September 2022). The bibliometric methodology (Fig. S1(a)) was performed during the 1995-2021 period by searching "article titles, abstracts, and keywords".


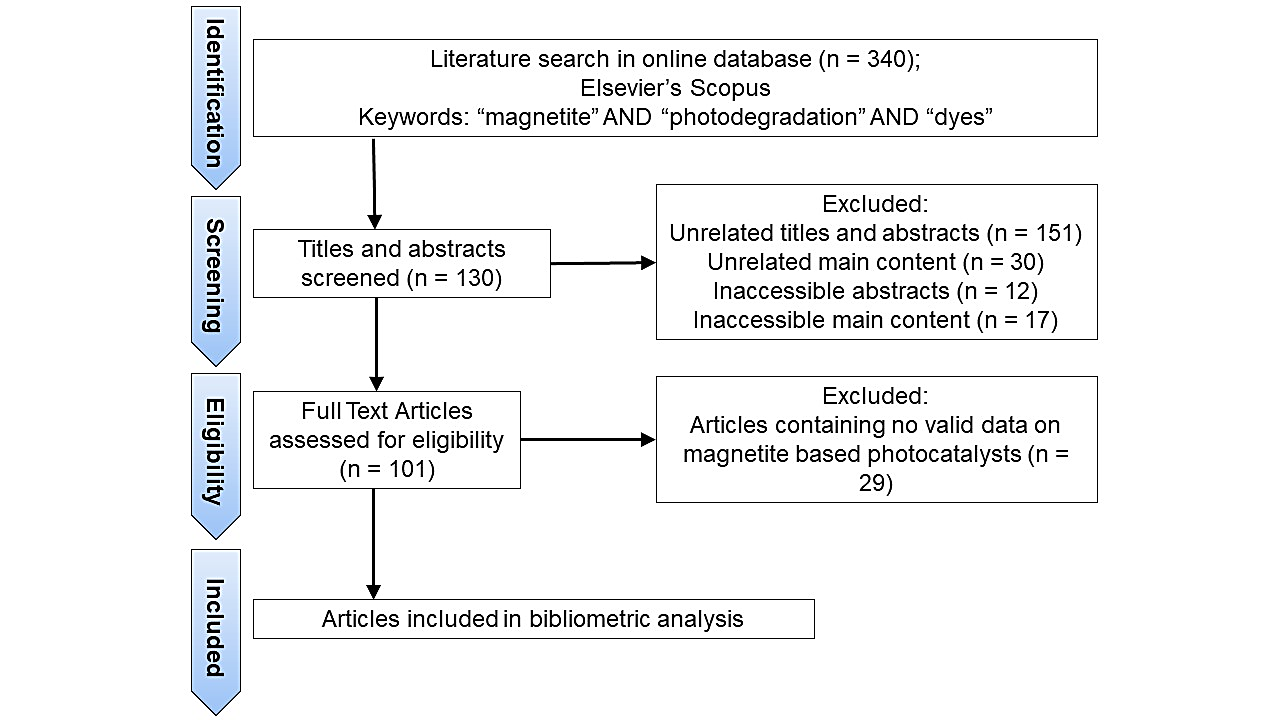


**(a)**


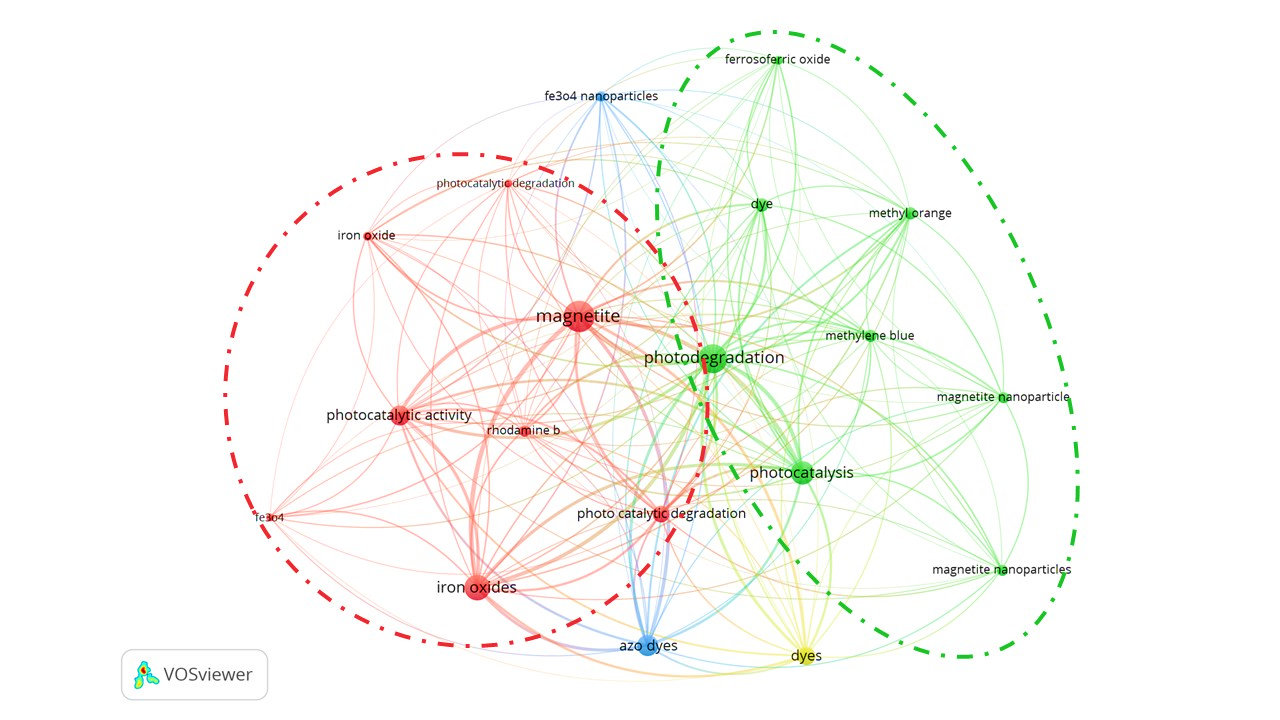


**(b)**


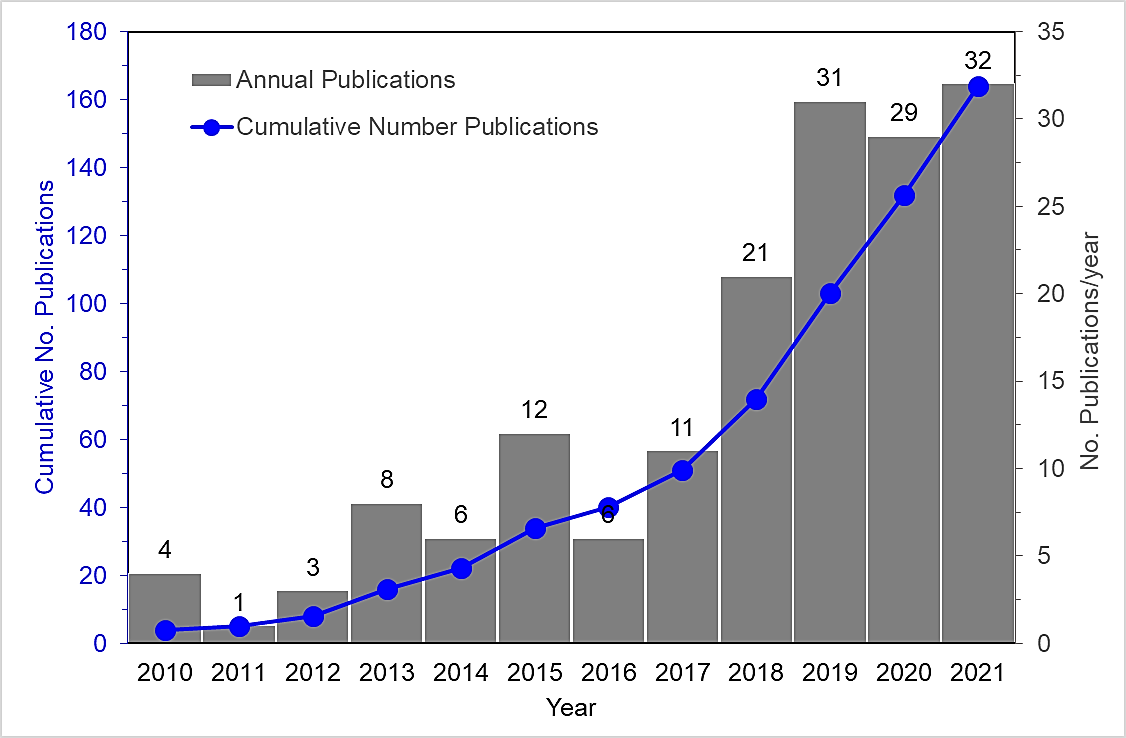


**(c)**


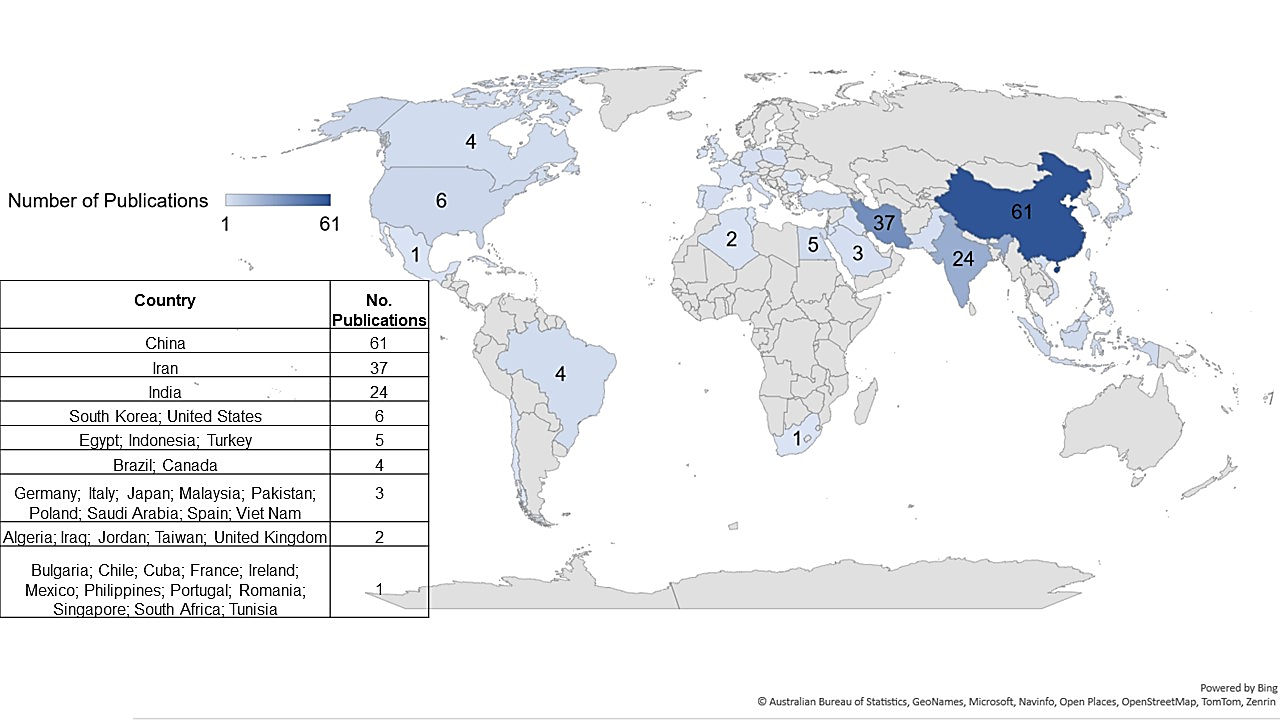


**(d)**

Fig. S1: (a) publications inclusion and exclusion criteria for bibliometric data, (b) co-word network of publications, (c) number of publications and cumulative pattern, and (d) worldwide map of research

The keyword analysis method was employed to generate a co-word network using VOSviewer 1.6.18 software (Zhao et al. 2020). A total of 1971 keywords were identified by the software, which were reduced to 214 keywords by setting the minimum keyword occurrence per publication at 5. This step was followed by the selection of all keywords related to photo-degradation using magnetite, *viz*., “azo dyes”, “dye”, “dyes”, “Fe_3_O_4_”, “Fe_3_O_4_ nanoparticles”, “ferrous/ferric oxide”, “iron oxide”, “iron oxides”, “magnetite”, “magnetite nanoparticle”, “magnetite nanoparticles”, “methylene blue”, “methyl orange”, “photocatalysis”, “photocatalytic activity”, “photocatalytic degradation”, “photo catalytic degradation”, “photodegradation”, and “rhodamine b”; and discarding the undesired keywords.

These procedures yielded a total of 19 keywords, which were then used to generate the co-word network shown in Fig. S1(b). The thickness of their interconnecting lines gives the strength of the relationships between various keywords. Moreover, the keyword occurrence frequency is noted by the size of the keyword node. Two main clusters could be defined from the generated co-word network, where each cluster contains strongly correlated keywords. The Red Cluster includes mostly keywords relating to magnetite and iron oxides and photocatalytic activity. This cluster demonstrates that researchers have recently taken an interest in exploring iron oxide nanomaterials for the photodegradation of dye-laden wastewater. This interest could be alluded to the essential factors and characteristics, such as desirable band gap energies and stability (Warsi et al. 2021), and the magnetic properties of iron oxides. These oxides include several compounds, such as magnetite (Fe_3_O_4_) and hematite (Fe_2_O_3_). The Green Cluster contains keywords associated with photodegradation and dyes, including “dye”, “methyl orange” and “methylene blue”. As dyes cause significant water pollution (Chowdhury et al. 2016), researchers have focused on the photodegradation of textile industrial effluents. Moreover, methyl orange and methylene blue were the common dyes used in clothing and textile industries and paper and leather manufacturing. The Blue Cluster is limited to "azo dyes" because the elimination of these dyes is a crucial task to avoid carcinogenic and mutagenic risks.

Fig. S1(c) shows a gradual increase in the number of publications defined by the same search string described previously, as shown in Fig. S1(a)) during the 2010-2017 period. After 2017, this increased approximately two-fold. The researchers took an active interest in understanding the performance of magnetite as a photocatalyst for removing dyes from wastewater. Moreover, the 2019-2021 phase covered the studies that focused on discovering innovative methods to separate photocatalyst materials from the aqueous solutions. This 2-year period maintained a steady publication rate, with an average number of about 30 publications per year, resulting in a cumulative number of 164 publications by 2021.

A map of worldwide research was generated using the same SCOPUS assessment (Fig. S1(d)), identifying a total of 35 countries. The majority of publications made during the 2010-2021 period were assigned to China, followed by Iran and India. China is one of the countries producing the most colored cotton, woolen and silk fabrics and chemical fibers, making the textile industry a major contributor to the country's economic growth. Similarly, the economic status of Iran and India depends largely on exporting and manufacturing textile products and machinery. Egypt, the United States of America, South Korea, Turkey, Japan, Germany, Canada, and Indonesia also participated in a good number of publications (Fig. S1(d)). The researchers of these countries focus on minimizing the waste generated from the textile mills and protecting the environment from the disposal of inorganic salts, dyes, and heavy metals. Moreover, most of these countries encouraged the application of magnetite-based composite photocatalysts for treating textile effluents.

# **Dyes: types and structures**

## **Azo Dyes**

The chemical structure of azo dyes is composed of one (mono-) or multiple (di-, tri-, etc.) azo functional groups (-N=N-) (Said et al. 2020). This chemical group is responsible for making nonbiodegradable, recalcitrant, and persistent azo dyes. This category includes acid, basic, reactive, and disperse dyes. As such, azo dyes have been identified as the largest group of colorants in the commercial sector (textiles, food, and clothing), representing more than 50% of industrial dyes pollution (Biswas et al. 2022). Based on their constitution, azo dyes can be identified by the azo chromophore and its attachment to at least one aromatic group (Kumar et al. 2021). When subjected to photocatalytic degradation, the azo bond (-N=N-) is initially attacked/dissociated, resulting in dye decolouration. The dissociation of this azo bond forms one or multiple aromatic amines, which are further degraded by a series of reduction and oxidation reactions. These reactions are accompanied by releasing less toxic aromatic acids, followed by aliphatic acids until the formation of much simpler molecules, such as CO_2_ and H_2_O.

## **Acid Dyes**

Acid dyes can perform their required functions, *viz*., to dye natural protein fibers (wool and silk), and synthetic polyamide (nylon), at relatively low pH conditions. The acid dyes are sodium salts of organic acids, and hence, their related wastewater contains high COD and solids contents. The chemical structure of acid dyes is classified into anthraquinone, azo, and triphenylmethane. When dissolved in water, acid dyes are dissociated to generate negatively charged (anionic) dye molecule ions (Chiu et al. 2019). The acid dye structure is distinguished by its attachment to one or two highly water-soluble sulfonated groups (–SO_3_Na), facilitating dye diffusion and migration into the fiber. Acid dyes resist conventional biodegradation and chemical oxidation because of their complex aromatic structures; hence, multiple acidic compounds are released as secondary pollutants under photodegradation.

## **Basic dyes**

Basic dyes are considered water-soluble cationic dyes because of two major reasons (i) they are salts of organic bases, and (ii) under dyeing conditions, the dye molecules give coloured cations (e.g., positively charged amine group) (Ali 2006). This group of positive dyes forms a strong electrostatic “attraction” with the negatively charged cellulose fibre (e.g., negatively charged SO_3_H groups) (Kiron 2013). The attached basic group, such as –NH_2_, –NHR, –NR_2_, and their salts, dissociate in the solution matrix to give the dye molecule an overall positive charge (cationic molecule). Triarylmethane, xanthene, and diphenylmethane are the main classes of basic dyes. Basic dyes usually tend to have low levelling properties, i.e., they have slow migration and rarely spread or dye the substrate (e.g., leather, paper, or textile fibers) during application. As such, basic dyes are combined with cationic retarding agents over a selected temperature range to overcome levelling difficulties in dyeing (Kiron 2013; Richards 2012).

## **Vat Dyes**

The name “Vat dyes” was coined because this group of dyes could be formed by fermentation processes (e.g., fermenting leaf pulp) that occur in large vessels known as *‘vats’.* Vat dyes can be identified by the inclusion of one or more pairs of carbon atoms doubly bonded to an oxygen atom. These dyes belong to a chromophore group, called the carbonyl group (Kumar et al. 2021). Although vat dyes are water-insoluble pigments, their solubility under alkaline conditions is considerable. Effective reduction and solubilization of vat dyes follow two major steps (i) reducing the dye compound under strongly alkaline conditions (e.g., sodium hydroxide and sodium hydrosulfite) into a soluble and substantive form (leuco form) by a vatting process (Kumar et al. 2021; Richards 2012), and (ii) bringing out (re-solubilize) the colour of the dyes through subjecting leuco compound on the fabric fibres to oxidation processes either in the air or by adding an oxidizing agent (Ali 2006). Vat dyes are not a preferable choice for the cotton parts, but they can be used in cellulosic fibre production.

## **Reactive Dyes**

Reactive dyes comprise chromophore groups, and they can be distinguished by the presence of a reactive group, such as dichlorotriazine, sulphatoethylsulphone, difluorochloropyrimide, and dichloroquinoxaline, in their structure. The reactive chemical function encourages the creation of a strong covalent bond with the fibers, giving bright colours and excellent fastness properties (Richards 2012). Hence, reactive dyes are most commonly used in dyeing and printing cellulosic fibres, such as cotton and viscose (Chattopadhyay 2011). Some reactive dyes could form complexes with Cu, Cr, and Ni, generating toxic heavy metals during photodegradation (Uddin and Rehman 2018).

## **Disperse Dyes**

Disperse dyes are a commonly azo chromophore group, and their application requires the inclusion of a carrier in the dyeing bath to enable dye diffusion into the substrate (Kumar et al. 2021; Said et al. 2020). Disperse dyes display low water solubility, non-ionic character, and poor wet-fastness properties. As such, the dye application process is usually performed at high temperatures to facilitate the dye’s infusion into the substrate fibres (Said et al. 2020). They are commonly used to dye thermoplastic hydrophobic materials or fibres, such as acrylics, polyester, and nylon (Ali 2006; Moody and Needles 2004). Disperse dye is non-polar, i.e., they do not possess an overall anionic or cationic charge (Moody and Needles 2004). Disperse azo dyes are featured by their recalcitrant and non-biodegradable nature, and they undertake no noticeable chemical changes during dyeing (Kiron 2021).

## **Sulphur Dyes**

Sulfur dyes are inexpensive, highly coloured, available, and easy to use in paste or powder forms. These dyes represent sulfur-containing molecules (high molecular weight dyes) mainly employed for dyeing cellulosic fibers (Chakraborty 2011; Kiron 2021). Sulfur dyes are synthesized by the thermal treatment (melting or boiling) of organic compounds containing amino or nitro groups with Na-polysulphide and Sulfur (El-Sikaily et al. 2012). Similar to azoic and disperse dyes, sulphur dyes are water-insoluble, i.e., they are initially reduced into a leuco form (sodium-derived leuco) using a reducing agent (e.g., Na_2_S alkaline medium) and then re-converted into their original form by oxidation (Uddin and Rehman 2018). This re-oxidation process facilitates a strong adherence of the Sulfur dye to the substrate fibres (Kiron 2021). During the photodegradation of Sulfur black dye, the dimethyl *n*-sulfane bridge linkage in the dye structure was initially cleaved, followed by oxidation of the generated intermediates until obtaining simpler degradation by-products (Touati et al. 2019).

# **Degradation by-products**

The azo (-N=N-) bond in the azoic dye structure is extremely active and its cleavage occurs by an efficient electron transfer to produce ^•^OH and accumulation of the photogenerated holes (Rauf and Ashraf 2009). In a similar way, the azo bonds can undergo reduction reactions by taking up the photogenerated electrons. The breaking or cleavage of the azo bond in azoic dyes is usually the first reaction that occurs in the dye degradation process, resulting in the decolourization of the dye solution. However, this reaction does not directly eliminate the solution TOC content. This cleavage further results in the formation of intermediate aromatic compounds which get further degraded into aliphatic compounds possessing relatively lower molecular masses. Finally, the mineralization of such compounds into much simpler elements and ions, such as organic acids, CO_2_, SO_4_^2−^, NH_4_^+^ and NO_3_^−^, occurs to form complete dye removal (Rauf and Ashraf 2009).

Table S1: Applications of the treated effluents in the textile industries for preparing an amount of fabric= 400 Kg

| Process | Water utilization | Volume of water (m^3^) |
| --- | --- | --- |
| Scouring (pre-treatment) | Water is used with alkali, detergent, and sequestrant to eliminate impurities from the cotton at approximately 100°C | 2.6 |
| Bleaching (pre-treatment) | Water is used to prepare sodium hypochlorite or hydrogen peroxide solutions to whiten fabric | 13.0 |
| Neutralizing (pre-treatment) | Water is used to prepare an acid (usually acetic acid) for adapting the solution pH, because the effluents of scouring and bleaching are highly alkaline. This is required to avoid dye reaction with the fabric in the process. | 5.2 |
| Dyeing | The dyeing process require large amount of water to prepare salt and alkaline conditions for fixation onto the fabric. Treated water could also be utilized to prepare some polyester and polyester blends for dyeing. | 2.8 |
| Rinsing (post-treatment) | In this process, large amounts of treated textile effluents are added to the dye-bath in a concurrent water flowing system. This process is employed to washout excess chemicals (e.g. unfixed dye) from the fabric. | 31.2 |
| Softening and finishing (post-treatment) | After multiple rinsing and washing steps, water is added to a mixture of softeners and other chemicals and applied to knitted cotton fabrics. The synthesised mixture offers the fabric its aesthetic, chemical and mechanical properties. These features include flame retardant, mildew resistance, and wrinkle resistance. | 2.6 |

**References**

Ali A (2006) Department of Chemistry A.M.U., Aligarh B.Sc. (HONS.) VI Semester (C.B.C.S) Organic Chemistry – AE (CHB 67Z.

Biswas S, Ghosh S, Maji S, Das S, Roy S, Bhattacharjee R, Mitra P, Malik S, Dey A (2022) Mechanistic Aspect of the Dye Degradation Using Photocatalysts. In: Dave S, Das J (Eds.) Trends and Contemporary Technologies for Photocatalytic Degradation of Dyes, pp 247-284. Retrieved from https://doi.org/10.1007/978-3-031-08991-6_10

Chakraborty J (2011). Sulphur dyes. Handbook of Textile and Industrial Dyeing: Principles, Processes and Types of Dyes, Sawston, United Kingdom

Chattopadhyay D (2011) 4 - Chemistry of dyeing. (M. Clark, Ed.) Handbook of Textile and Industrial Dyeing, 1, 150-183. Retrieved from https://www.sciencedirect.com/science/article/pii/B9781845696955500040

Chiu Y, Chang T, Chen C, Sone M, Hsu Y (2019). Mechanistic insights into photodegradation of organic dyes using heterostructure photocatalysts. Catalysts, 9(5). https://doi.org/10.3390/catal9050430

Chowdhury S, Khan N, Kim GH, Harris J, Longhurst P, Bolan N (2016) Chapter 22 - Zeolite for Nutrient Stripping from Farm Effluents. In: Prasad M, Shih K (Eds.) Environmental Materials and Waste, pp 569-589.

El-Sikaily A, Khaled A, El Nemr A (2012) Textile Dyes Xenobiotic and Their Harmful Effect. In: El Nemr A (Ed) Non-Conventional Textile Waste Water Treatment, pp 31-64.

Kiron M (2013). Properties, Classification and Application of Basic Dyes. Textile Learner. https://textilelearner.net/basic-dyes-properties-classification/. Accessed 26 November 2023

Kiron M (2021a) Disperse Dyes: Properties, Classification, Dyeing & Printing Method. Textile Learner. https://textilelearner.net/disperse-dyes-dyeing-and-printing-method/. Accessed 26 November 2023

Kiron M (2021b) Reactive Dyes: Classification, Dyeing Mechanism, Application & Stripping. Textile Learner. https://textilelearner.net/reactive-dyes-classification-dyeing-mechanism/. Accessed 26 November 2023

Kumar A, Dixit U, Singh K, Gupta S, Beg M (2021) Structure and Properties of Dyes and Pigments. In: Papadakis R (Ed.) Dyes and Pigments. https://doi.org/10.5772/intechopen.97104

Moody V, Needles H (2004) 15 - Color, Dyes, Dyeing, and Printing. (V. Moody, & H. Needles, Eds.) Tufted Carpet, 155-175. Retrieved from https://www.sciencedirect.com/science/article/pii/B9781884207990500166

Rauf M, Ashraf S (2009). Fundamental principles and application of heterogeneous photocatalytic degradation of dyes in solution. J Chem Eng 151:10-18. https://doi.org/10.1016/j.cej.2009.02.026

Richards P (2012). 17 - Dye types and application methods. (J. Best, Ed.) Colour Design, 471-496. Retrieved from https://www.sciencedirect.com/science/article/pii/B9781845699727500175

Said B, Souad MR, Ahmed E (2020) A review on classifications, recent synthesis and applications of textile dyes. Inorg Chem Commun 3:107891. https://doi.org/10.1016/j.inoche.2020.107891

Touati A, Jlaiel L, Najjar W, Sayadi S (2019). Photocatalytic degradation of sulfur black dye over Ce-TiO_2_ under UV irradiation: removal efficiency and identification of degraded species. Euro-Mediterr J Environ Integr 4(1). https://doi.org/10.1007/s41207-018-0086-5

Uddin M, Rehman Z (2018) Application of Nanomaterials in the Remediation of Textile Effluents from Aqueous Solutions. Nanomaterials in the Wet Processing of Textiles, 135-161. Retrieved from https://onlinelibrary.wiley.com/doi/abs/10.1002/9781119459804.ch4

Warsi M, Shaheen N, Sarwar M, Agboola P, Shakir I, Zulfiqar S (2021) A comparative study on photocatalytic activities of various transition metal oxides nanoparticles synthesized by wet chemical route. Desalin Water Treat 211:181-195. https://doi.org/10.5004/dwt.2021.26463

Zhao L, Dai T, Qiao Z, Sun P, Hao J, Yang Y (2020). Application of artificial intelligence to wastewater treatment: A bibliometric analysis and systematic review of technology, economy, management, and wastewater reuse. Process Saf Environ Prot 133:169-182. https://doi.org/10.1016/j.psep.2019.11.014
